# Small-Molecule Proteomimetic Inhibitors of the HIF-1α–p300 Protein–Protein Interaction

**DOI:** 10.1002/cbic.201400009

**Published:** 2014-04-29

**Authors:** George M Burslem, Hannah F Kyle, Alexander L Breeze, Thomas A Edwards, Adam Nelson, Stuart L Warriner, Andrew J Wilson

**Affiliations:** [a] School of Chemistry, University of Leeds Woodhouse Lane, Leeds LS2 9JT (UK) E-mail: J.Wilson@leeds.ac.uk; [b] Astbury Centre for Structural Molecular Biology, University of Leeds Woodhouse Lane, Leeds, LS2 9JT (UK); [c] School of Molecular and Cellular Biology, Faculty of Biological Sciences, University of Leeds Woodhouse Lane, Leeds LS2 9JT (UK); [d] Protein Structure and Biophysics, Discovery Sciences, AstraZeneca R&D Alderley Park, Cheshire, SK10 4TG (UK)

**Keywords:** helix mimetics, hypoxia, inhibitors, peptidomimetics, protein–protein interactions

## Abstract

The therapeutically relevant hypoxia inducible factor HIF-1α–p300 protein–protein interaction can be orthosterically inhibited with α-helix mimetics based on an oligoamide scaffold that recapitulates essential features of the C-terminal helix of the HIF-1α C-TAD (C-terminal transactivation domain). Preliminary SAR studies demonstrated the important role of side-chain size and hydrophobicity/hydrophilicity in determining potency. These small molecules represent the first biophysically characterised HIF-1α–p300 PPI inhibitors and the first examples of small-molecule aromatic oligoamide helix mimetics to be shown to have a selective binding profile. Although the compounds were less potent than HIF-1α, the result is still remarkable in that the mimetic reproduces only three residues from the 42-residue HIF-1α C-TAD from which it is derived.

An emerging goal in cancer chemotherapy is to target metabolic and cellular processes that enable the survival and growth of tumours.[1] The transcription factor, hypoxia inducible factor (HIF), plays a central role in the cellular response to hypoxia. HIF exists as three isoforms (1–3), with HIF-1 and, to a lesser extent, HIF-2 identified as drivers of tumour growth.[2] HIF-1 is a heterodimer made up of two subunits: HIF-1α and HIF-1β (HIF-1β is also referred to as aryl hydrocarbon receptor nuclear translocator, ARNT). Under normoxic conditions HIF-1α is rapidly degraded through an oxygen-dependent process with the von Hippel–Lindau protein (pVHL) playing a dominant role.[3] Under hypoxic conditions, however, the protein HIF-1α is stabilised and translocated to the nucleus, where it forms heterodimers and recruits transcriptional coactivator proteins such as p300,[4, 5] and this leads to the hypoxic response cascade. This results in expression of multiple genes (e.g., VEGF) that participate in angiogenesis, various metabolic processes and cell proliferation and survival. Solid tumours develop rapidly, and oxygen supply diminishes; cancerous cells thus exploit the hypoxic response pathway to initiate resupply of the tumour with oxygen through formation of new vasculature.

Targeting the HIF pathway has therefore become the focus of efforts to develop small-molecule inhibitors.[2] However, HIF's function as a transcription factor is exerted through protein–protein interactions (PPIs). PPIs are considered challenging targets for small-molecule ligands, given that the target surfaces for competitive inhibition are typically large and less well defined than conventional small-molecule binding “pockets”.[6, 7] Despite this, several approaches to target the HIF pathway have been described. Inhibitors of the VHL–HIF-1α interaction (identified through fragment approaches),[8] polyamide inhibitors of HIF-1–DNA binding,[9] cyclic peptide inhibitors of HIF-1 heterodimerization (identified through screening of genetically encoded cyclic peptide libraries),[10] orthosteric inhibitors of HIF-1β–coactivator interactions[11] and allosteric small molecules that attenuate HIF-2 dimerization[12] have all been described.

Inhibition of the HIF-1α–p300 interaction[13] also represents an attractive approach for modulation of HIF-1α; identification of selective and specific probe molecules should facilitate studies of the HIF pathway and might be advantageous in terms of developing therapies. Natural products, such as chetomin (**1**, Figure 1 A),[14] and other epidithioketopiperazine (ETP)-containing small molecules[15] have been reported to act as HIF-1α–p300 inhibitors; however, the ETP motif ejects structurally important zinc from p300, and so these compounds are unlikely to act as selective and specific inhibitors.[16] Similarly, small molecules[17–19] such as compound **2** (Figure 1 A),[18, 19] obtained by high-throughput screening in cellular assays, have been shown to down-regulate expression of HIF-dependent genes. However, it is unclear whether these molecules disrupt the HIF-1α–p300 interaction directly or prevent downstream expression in another way, such as preventing HIF-1α expression or HIF-1α–HIF-1β dimerization or by inhibiting necessary post-translational modifications.

**Figure 1:**
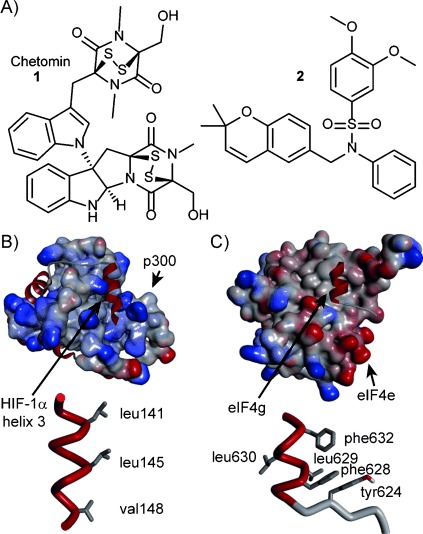
A) Structures of previously reported inhibitors: natural product chetomin (1)[13] and synthetic compound 2 reported by Van Meir.[14] B) NMR structure (PDB ID: 1L8C)[5] of p300 in complex with the C-terminal transactivation domain of HIF-1α (top) and excised C-terminal helix of HIF-1α showing key side chains (bottom). C) X-ray crystal structure (PDB ID: 2W97)[15] of the eIF4E–eIF4G complex (top) and the excised helix of eIF4G showing key side chains (bottom).

A published NMR structure (PDB ID: 1L8C) of the HIF-1α–p300 complex[5] (Figure 1 B) has shown that the HIF-1α C-terminal transactivation domain (C-TAD) adopts an α-helical conformation and wraps itself around the CH1 domain of p300 with the reported key residues on helices 2 and 3[20] displayed on one face, making them an obvious target for inhibition with designed ligands[21] such as constrained peptides,[22, 23] β-peptides[24] or helix mimetics.[25] Indeed, Arora and co-workers have described hydrogen-bond-surrogate stabilized helices[26, 27] that bind to p300 as evidenced by a variety of biophysical methods,[26, 27] down-regulate HIF-1α-inducible genes[26, 27] and suppress tumour growth in murine xenograft models of renal cell carcinoma.[26] Here we describe the first biophysically characterised small-molecule inhibitors of the HIF-1α–p300 interaction. We have employed a proteomimetic approach in which an aromatic oligoamide[28] was used to project side chains deemed essential to the PPI in a spatial orientation identical to that in the native helix.[29–34]

Using the NMR structure (PDB ID: 1L8C) of the HIF-1α–p300 complex,[4] we designed and synthesised compounds intended to mimic the key functionalities and spatial orientation of the C-terminal helix (helix 3) of HIF-1α. Helix 3 presents hydrophobic residues along one face of the helix at the *i*, *i*+4 and *i*+7 positions (Figure 1 B). We initially prepared the two compounds **3** and **4** (Scheme 1, see the Supporting Information for synthesis and characterisation), designed to act as direct mimics of this helix, by the modular oligobenzamide synthetic methodology we have previously reported.[28, 29] The 3-*O*-alkylated oligobenzamide scaffold was chosen due to the robust modular solution[28, 35] and solid-supported[36] syntheses we have developed, the ability to adopt the desired conformation in solution[28, 35] and its proven ability to inhibit α-helix-mediated PPIs.[29, 30, 32] Compounds **3** and **4** (Scheme 1) recapitulate the Leu, Leu and Val side chains in both the matched and the mismatched N-to-C orientation with respect to the natural helix; it is noteworthy that prior molecular dynamics simulations suggested that both parallel and antiparallel orientations can adopt stable bound conformations with target proteins.[37]

**Scheme 1:**
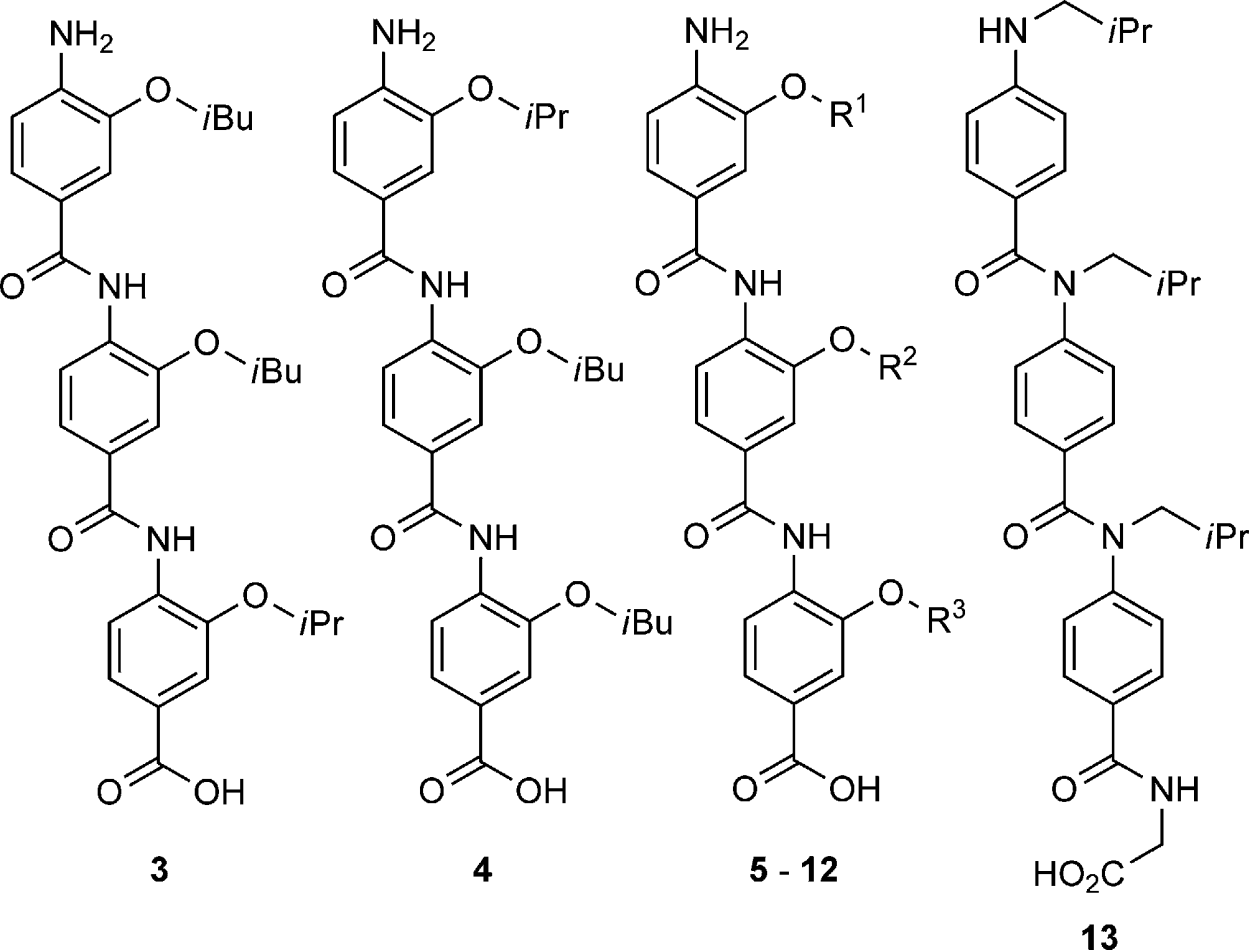
Structures of aromatic oligoamides.

The compounds **3** and **4** were tested in a fluorescence anisotropy competition assay with a 42-residue peptide derived from the C-TAD of HIF-1α, N-terminally labelled with FITC through an aminohexanoic acid linker, together with residues 330–420 of the CH1 domain of p300 (see the Supporting Information for details of assay development, protein cloning, expression and purification).[38] Upon titration of the compounds, we observed decreases in anisotropy associated with the disruption of the interaction in a similar manner to the decrease observed upon titration of the unlabelled peptide (Figure 2 A). In our assay the IC_50_ value of the unlabelled peptide is 0.23 μm, whereas compounds **3** and **4** give IC_50_ values of 9.19 and 24.0 μm, respectively. The result is significant given that the 16-residue sequence of helix 3, the basis upon which compounds **3** and **4** were designed, was shown to be inactive in the fluorescence anisotropy assay (see the Supporting Information). We attempted to perform direct binding experiments by exploiting the tryptophan fluorescence present in p300; however, these were unsuccessful due to inner filter effects resulting from the intrinsic fluorescence of compound **3** (see the Supporting Information). Encouraged by these preliminary results, we prepared a small library of compounds with alternative side chains to probe the relative importance of the side chain size and polarity. The compounds and their IC_50_ values are summarised in Table 1.

**Figure 2:**
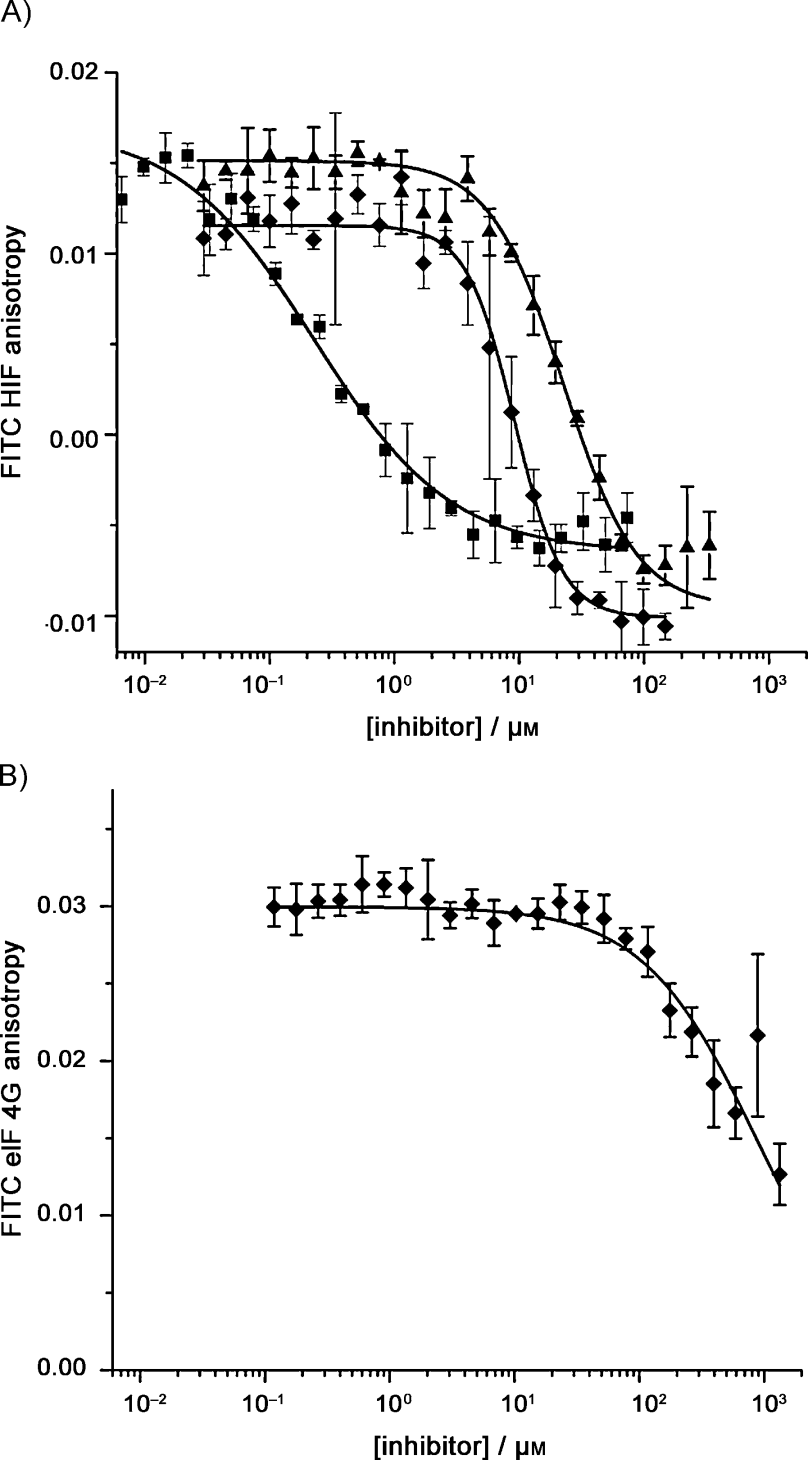
Fluorescence anisotropy competition assays for helix mimetics tested against different helix-mediated PPIs. A) FITC-HIF-1α fluorescence anisotropy competition assay data for the unlabelled HIF-1α (▪), compound 3 (⧫) and compound 4 (▴; 40 mm sodium phosphate buffer pH 7.5, 80 nm FITC-HIF-1α, 100 nm p300). B) FITC-eIF4G fluorescence anisotropy competition assay data for compound 3 (diamonds; 40 mm sodium phosphate buffer pH 7.5, 80 nm FITC-eIF4G, 3 μm eIF4E). Error bars represent standard deviations of the means (*n*=3).

**Table 1:** Structures and IC_50_ values for compound library

Compound	R^1^	R^2^	R^3^	IC_50_ [μm]
helix 3 peptide	*Ac-GTEELLRALDQVNAAG-NH_2_*			inactive^[c]^
**3**	*i*Bu	*i*Bu	*i*Pr	9.2±0.9
**4**	*i*Pr	*i*Bu	*i*Bu	24±1.6
**5**	Me	*i*Pr	*i*Bu	216±16^[a]^
**6**	*i*Bu	*i*Bu	*i*Bu	9.8±1.3
**7**	*i*Bu	*i*Pr	*i*Bu	13±1.5
**8**	benzyl	benzyl	benzyl	56±6.0
**9**	*i*Pr	*i*Pr	*i*Pr	39±4.0
**10**	*i*Bu	*i*Pr	*i*Pr	17±0.7
**11**	benzyl	*i*Pr	*i*Pr	20±0.8
**12**	2-hydroxyethyl	*i*Pr	*i*Pr	416±64^[a]^
**13**^[b]^	*i*Pr	*i*Pr	*i*Pr	inactive^[c]^

[a] Estimated IC_50_. [b] N-alkylated scaffold. [c] Up to >250 μm.

Overall, the most potent compound identified was the exact mimic of the helix side chains with the same N-to-C sequence of side chains as the native peptide sequence. The next most potent compound, **6**, had matched top and bottom residues but a very small difference (*i*Bu to *i*Pr) in the central position. Incorporation of larger aromatic side chains (e.g., **8**), in all positions had a detrimental impact upon inhibitory potency. Additionally, our structure–activity relationship studies suggest that the R^1^ side chain makes important contacts within the binding cleft through solvophobic effects. This is backed up by the reduced binding of **5** (because a methyl group was shown to be insufficient to promote inhibition of the interaction) and **12** (because the introduction of polar functionality into a hydrophobic binding site is disfavoured). It should also be noted that the nitroester precursor to **3** had an IC_50_ greater than 1 mm; this suggests that the amine, the acid or both impart significant affinity along with an improvement in solubility.

To gain further insight into the nature of molecular recognition, docking simulations were performed (Figure 3). These docking studies identified a binding pose that would be expected on the basis of the pharmacophore upon which the helix mimetics were based. Each of the three hydrophobic side chains on **3** is matched to the position it mimics in the helix 3 sequence of the HIF-1α C-TAD. Notably, the central R^2^ isobutyl side chain sits in a hydrophobic pocket defined by His20, Leu17 and Leu16 of p300.

**Figure 3:**
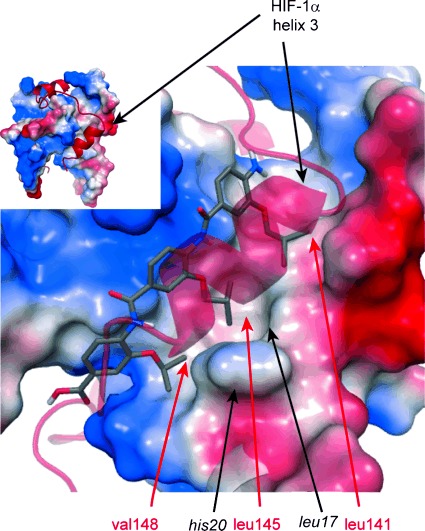
Molecular docking studies on compound 3; proposed binding mode of compound 3 in the HIF-1α C-TAD helix 3 binding cleft with the native peptide in transparent red. Inset: structure of HIF-1α–p300 complex (PDB ID: 1L8C).

Considering the range of peptidomimetic compounds reported in the literature, we sought to compare the activities of the *O*-alkylated compounds with the activities of compounds derived from an alternative scaffold. Oligobenzamide scaffolds featuring alkylation on the amide nitrogen have previously been shown to be capable of mimicking an α-helix.[31, 39] Hence, compound **13** was tested in the fluorescence anisotropy competition assay as a comparison with compound **6**, which features the same side chains. No inhibition of the HIF-1α–p300 PPI was observed with **13** up to a concentration of 250 μm. This suggests that the position of functionality within the scaffold and/or its conformation is crucial for the activities of the inhibitors reported here.

Having developed oligoamide inhibitors, we also sought to compare our compounds with those reported in the literature. A modified synthesis of compound **2** was thus developed (see the Supporting Information), and **2** was tested in our fluorescence anisotropy assay. Although **2** had been shown by others to have an IC_50_ of 0.65 μm in a cellular reporter assay,[19] its solubility only permitted testing in our assay up to a maximum concentration of 25 μm; at these concentrations we observed no inhibition of the HIF-1α–p300 interaction; this suggests that this compound might modulate the HIF pathway through a different target or targets.

Finally, to ascertain whether the oligobenzamide compound has a selective binding profile, compound **3** was tested for inhibitory activity on another therapeutically relevant α-helix-mediated PPI—eukaryotic initiation factor 4E/4G (eIF4E/eIF4G; Figure 1 C).[40–42] In a fluorescence anisotropy competition titration (Figure 2 B; see the Supporting Information for details of the protein, peptide preparation and assay development), the oligobenzamide compound **3** was a weak inhibitor of this alternative α-helix-mediated PPI, with an IC_50_>1 mm, representing over 100-fold selectivity in favour of the HIF-1α–p300 interaction over the eIF4E/eIF4G interaction.

In summary, we have shown that the therapeutically relevant HIF-1α–p300 PPI can be orthosterically inhibited with α-helix mimetics based on an oligoamide scaffold that recapitulates essential features of the C-terminal helix of the HIF-1α C-TAD. These compounds were shown to act as selective inhibitors of this interaction, whereas preliminary SAR studies demonstrated the important role of side chain size and hydrophobicity/hydrophilicity in determining potency. Furthermore, these small molecules represent the first biophysically characterised HIF-1α–p300 PPI inhibitors and the first examples of aromatic oligoamide helix mimetics to be shown to have a selective binding profile. Although the compounds were shown to be two orders of magnitude less potent than HIF-1α, the result is still remarkable in that the mimetic reproduces only three residues from the 42-residue HIF-1α C-TAD from which it is derived. Crucially, the polypeptide reproducing only the 16-residue C-terminal helix 3 of HIF-1α C-TAD upon which the compound **3** was designed was shown to be inactive in this assay. Our on-going studies are focused upon further elucidating the binding mode of these compounds by other biophysical, biochemical and structural techniques and upon identifying inhibitors active in cells.

## Supporting Information

As a service to our authors and readers, this journal provides supporting information supplied by the authors. Such materials are peer reviewed and may be re-organized for online delivery, but are not copy-edited or typeset. Technical support issues arising from supporting information (other than missing files) should be addressed to the authors.

miscellaneous_information
