# Supplementary material for: Small-Molecule Proteomimetic Inhibitors of the HIF-1α–p300 Protein–Protein Interaction
Source: Chembiochem. 2014 Apr 29;15(8):1083–7. doi: 10.1002/cbic.201400009 (PMC4159589; doi:10.1002/cbic.201400009)
Supplement: Supplementary file 1 — miscellaneous_information [file cbic0015-1083-sd1.pdf]

CHEMBIOCHEM

Supporting Information

© Copyright Wiley-VCH Verlag GmbH & Co. KGaA, 69451 Weinheim, 2014

Small-Molecule Proteomimetic Inhibitors of the HIF-1α–p300 Protein–Protein Interaction

George M. Burslem,[a, b] Hannah F. Kyle,[b, c] Alexander L. Breeze,[d] Thomas A. Edwards,[b, c] Adam Nelson,[a, b] Stuart L. Warriner,[a, b] and Andrew J. Wilson*[a, b]

cbic_201400009_sm_miscellaneous_information.pdf

Contents

General Experimental 3

Monomer Preparation 4

Synthesis of Oligobenzamides 4

Library Synthesis 15

Synthesis of Compound **2** 20

Plasmids 22

Protein Expression 22

Peptides 25

Fluorescence Anisotropy Assays 27

Docking 33

Competition Assays Curves 34

Spectra of Final Compounds 39

References 44

GENERAL EXPERIMENTAL

All commercial solvents and reagents were used without further purification unless stated otherwise. All non-aqueous reactions were performed under an atmosphere of nitrogen and using anhydrous solvents. Water-sensitive reactions were performed in oven-dried glassware, cooled under nitrogen before use, or flame dried and cooled, under vacuum if stated. Solvents were removed under reduced pressure using a Büchi rotary evaporator. Ether refers to diethyl ether and petrol refers to petroleum spirit (b.p. 40-60 °C). Commercially available starting materials were obtained from Sigma–Aldrich or Alfa Aesar. Flash column chromatography was carried out using silica (35-70 μm particles) or alumina (neutral, Brockman activity 1), with crude reaction mixtures loaded in the initial solvent system or its least polar constituent. Thin layer chromatography was carried out on commercially available silica pre-coated aluminium plates (Kieselgel 60 F254, Merck) or commercially available alumina pre-coated glass plates (neutral, Brockman activity 1).

Proton and carbon NMR spectra were recorded on a Bruker Avance 500, Avance DPX300 or DRX500 spectrophotometer with an internal deuterium lock. Carbon NMR spectra were recorded with composite pulse decoupling using the waltz 16 pulse sequence. Chemical shifts are quoted in parts per million downfield of tetramethylsilane, and coupling constants (*J*) are given in Hz. NMR spectra were recorded at 300 K unless otherwise stated. Infra-red spectra were recorded using a Perkin–Elmer Spectrum One FT-IR spectrophotometer. Melting points were determined using a Griffin and George melting point apparatus and are uncorrected. Nominal mass spectrometry was routinely performed on a Waters-Micromass ZMD spectrometer using electrospray (+) ionization. Nominal and accurate mass spectrometry using electrospray ionisation was carried out by staff or the authors in the School of Chemistry using a Micromass LCT-KA111, Bruker MicroTOF or Bruker MaXis Impact TOF mass spectrometer.

Atom Numbering and Trimer Naming

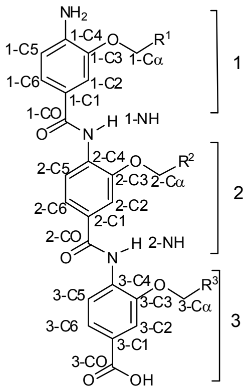

To simplify the numbering and NMR assignment of our trimers, we have devised a sequential nomenclature, where each of the monomer building blocks is considered separately. The monomers are numbered from 1 to 3 starting from the *N*-terminal. Within each monomer, the numbering is the same: the carbons from the aminobenzoic acid are numbered using the standard system (the aromatic carbon bearing the carboxylic acid is C1, the one bearing the amine is C4). Then, the lateral chain is numbered: the carbon attached to the oxygen is the Cα, and the numbering of the aliphatic part of the side chain continues with Cβ, etc. In the case of aromatic side chains, the

aromatic carbons are numbered CAr1, CAr2, etc. The numbering of the protons is based on the carbon numbering. To differentiate each individual carbon/proton, the monomer number is added as a prefix to the carbon/proton number representative examples are given above.

Synthesis of Oligobenzamides

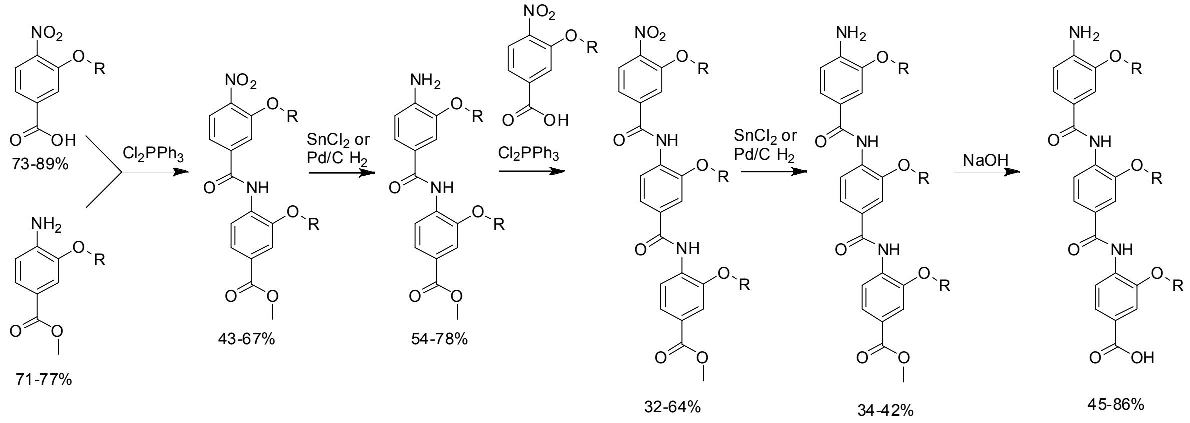

Standard Procedure A - Ester Hydrolysis

An aqueous sodium hydroxide solution (2 M, 1 mL per 100 mg of ester) was added to a solution of the ester in methanol (~5 mL per 100 mg of ester) and stirred at room temperature until the starting material had been consumed, as observed by TLC. The reaction mixture was concentrated by half *in vacuo* then adjusted to pH 3 by the addition of 1M HCl (aq.); the resulting precipitate was isolated by filtration, dried *in vacuo* and used without further purification in subsequent steps.

Standard Procedure B – Tin Mediated Nitro reduction

Tin (II) chloride dihydrate (5 equivalents) was added in one portion to a solution of the nitro compound in ethyl acetate (5 mL per 100 mg) and the reaction stirred at 50 °C under a calcium chloride drying tube for 24 hours. The reaction was then allowed to cool to room temperature and poured into 2 M sodium hydroxide solution (5 ml per 100 mg of starting material). The organic layer was separated, washed with 2 M sodium hydroxide solution (2 × 5 mL per 100 mg of starting material) and brine (5 mL per 100 mg of starting material), dried over magnesium sulphate and concentrated *in vacuo*. The residue was purified by flash column chromatography to give the desired compound.

Standard Procedure C – Nitro reduction by hydrogenation

Palladium on charcoal (10 %) was added against a flow of nitrogen to a solution of the nitro compound in methanol (10 mL per 100 mg) under a nitrogen atmosphere, the atmosphere was then replaced with hydrogen and the reaction stirred vigorously until complete by TLC (typically 2 hours).

The hydrogen atmosphere was vented and the reaction mixture filtered through a pad of Celite with methanol, concentrated *in vacuo* and purified by flash column chromatography.

Standard Procedure D - Coupling

Dichlorotriphenylphosphorane (4.5 equivalents) was added to a solution of nitro-acid compound (1.2 equivalents) in chloroform (5 mL per 100 mg of amine) and the reaction heated to reflux with stirring under nitrogen. After 2 hours at reflux, the amine ester compound (1 equivalent) was added as solution in chloroform (1 mL) and the reaction was heated to reflux for a further 24 hours. The reaction mixture was then concentrated *in vacuo* and partitioned between ethyl acetate (5 mL per 100 mg of amine) and H_2O (5 mL per 100 mg of amine). The organic layer was separated and washed with saturated aqueous sodium bicarbonate solution (5 mL per 100 mg of amine), dried over magnesium sulphate and concentrated *in vacuo*. The resulting residue was purified by flash column chromatography to give the desired compound.

MONOMER PREPARATION

Methyl 3-hydroxy-4-nitrobenzoate[1]

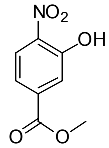

Following a literature procedure, *Para*-toluene sulphonic acid (*p*TSA) (0.415 g, 2.18 mmol, 20 mol%) was added to a suspension of 3-hydroxy-4-nitrobenzoic acid (2.00 g, 10.9 mmol) in methanol (40 mL) and the reaction heated at reflux for 18 hours. The reaction was concentrated *in vacuo* to afford a yellow solid which was then partitioned between H_2O (20 ml) and ethyl acetate (40 mL), the organic layer was washed with H_2O (2 × 20 mL) and saturated sodium bicarbonate solution (20 mL), dried over magnesium sulphate and concentrated *in vacuo* to yield the *title compound* as yellow needles (1.80 g, 87%); R_f 0.3 (20% EtOAc in Petrol); δ_H (500 MHz; $CDCl_3$) 10.50 (1H, s (broad), OH), 8.16 (1H, d, *J* 8.7, Ar-H), 7.83 (1H, d, *J* 1.6, Ar-H), 7.62 (1H, dd, *J* 8.7, 1.6, Ar-H), 3.95 (3H, s, OCH_3), δ_C (125 MHz; $CDCl_3$) 164.8, 154.6, 137.9, 135.8, 125.3, 121.6, 120.5, 52.9; *m/z* (ES) $[M+H]^+$ 197.

Methyl 3-isopropyloxy-4-nitrobenzoate[1]

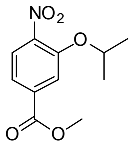

Potassium carbonate (2.1 g, 15.2 mmol) was added to a solution of methy-3-hydroxy-4-nitrobenzoate (0.60 g, 3.04 mmol) in DMF (5 mL), followed by 2-bromopropane (0.40 ml, 4.26 mmol)

and the reaction was stirred at 50 °C. After 20 hours the reaction was diluted with ethyl acetate (50 mL), washed with H_2O (2 × 50 mL) and brine (50 mL), dried over magnesium sulphate and concentrated *in vacuo* to give a pale yellow oil. The resulting residue was purified by flash column chromatography eluting with 1:1 Petrol–DCM to give the *title compound* as a yellow oil which crystallized on standing to give yellow needles (0.396 g, 54%), m.p. 54-56 °C (DCM); R_f 0.3 (1:1 Petrol–DCM); ν_{max}/cm^{-1} (solid state) 3437, 3119, 2990, 2516, 2159; δ_H (500 MHz; $CDCl_3$) 7.70 (2H, m, Ar-H), 7.59 (1H, dd, *J* 8.4, 1.4, Ar-H), 4.74 (1H, spt, *J* 6.2, $C_\alpha H$), 3.91 (3H, s, OCH_3), 1.36 (6H, d, *J* 6.2, $C_\beta H_3$ and $C_\gamma H_3$); δ_C (125 MHz; $CDCl_3$) 165.2, 150.6, 143.6, 134.4, 124.9, 121.0, 116.8, 72.9, 52.6, 21.6; HRMS *m/z* (ESI) Found 262.0686. $C_{12}H_{15}NO_5$ requires $[M+Na]^+$ 262.0691.

Methyl 3-(2-methyl-propyloxy)-4-nitrobenzoate[2]

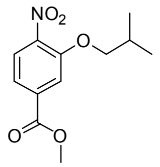

Potassium carbonate (4.20 g, 30.50 mmol) was added to a solution of methy-3-hydroxy-4-nitrobenzoate **2** (1.20 g, 6.09 mmol) in DMF (10 mL), followed by 1-bromo-2-methylpropane (0.93 mL, 8.52 mmol) and the reaction was stirred at 50 °C. After 20 hours a further aliquot of 1-bromo-2-methylpropane (0.93 mL, 8.52 mmol) was added and the reaction stirred at 50 °C for a further 24 hours. After this time the reaction was diluted with ethyl acetate (100 ml), washed with H_2O (2 × 100 mL) and brine (100 mL), dried over magnesium sulphate and concentrated *in vacuo* to an orange oil which crystallised on standing. The resulting mixture was purified by flash column chromatography eluting with 1:1 Petrol–DCM to give the *title compound* (0.964 g, 63%) as a yellow crystalline solid; m.p. 69-71 °C (from DCM–Petrol); R_f 0.3 (1:1 Petrol–DCM); ν_{max}/cm^{-1} (solid state) 3101, 2469, 2159, 1727; δ_H (500 MHz; $CDCl_3$) 7.78 (1H, d, *J* 8.2, Ar-H), 7.69 (1H, s, Ar-H), 7.62 (1H, d, *J* 8.2, Ar-H), 3.94 (3H, s, CO_2CH_3), 3.90 (2H, d, *J* 6.3, $C_\alpha H_2$), 2.13 (1H, sept, *J* 6.7, $C_\beta H$), 1.03 (6H, d, *J* 6.7, $C_\gamma H_3$ and $C_\delta H_3$); δ_C (125 MHz; $CDCl_3$) 165.1, 151.9, 142.3, 134.6, 125.7, 125.0, 120.9, 115.3, 52.6, 28.1, 18.8 (2C); *m/z* (ESI) 276.1 (100%, $[M+Na]^+$); HRMS Found: 276.0835; $C_{12}H_{15}NO_5$ requires $[M+Na]^+$ 276.0842.

Methyl 3-[2-(methoxymethoxy)ethoxy]-4-nitrobenzoate[2]

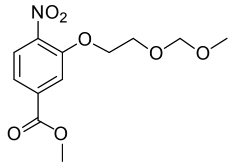

1-Bromo-2-(methoxymethoxy)ethane (0.41 mL, 3.55 mmol) was added to a solution of methyl 3-hydroxy-4-nitrobenzoate (0.50 g, 2.54 mmol) and potassium carbonate (1.70 g, 12.7 mmol) in DMF (10 mL) and heated to 50 °C for 24 hours, allowed to cool and partitioned between EtOAc (20 mL) and H_2O (20 mL). The organic layer was separated and washed with H_2O (20 mL) and brine (20 mL),

dried over magnesium sulphate, concentrated *in vacuo* to give a yellow solid and purified by column chromatography eluting with CH_2Cl_2 to give the desired product as a pale yellow solid (209 mg, 29%). m.p. 58-60 °C (CH_2Cl_2); R_f 0.3 (CH_2Cl_2); ν_{max}/cm^{-1} (Solid state) 2966, 2942, 2891, 1726; δ_H (500 MHz; $CDCl_3$) 7.83 (1H, d, *J* 8.2, Ar-H), 7.77 (1H, s, Ar-H), 7.69 (1H, d, *J* 8.2, Ar-H), 4.70 (2H, s, CH_2), 4.35 (2H, t, *J* 4.6, CH_2), 3.96 (3H, s, CO_2Me), 3.94 (2H, t, *J* 4.6, CH_2), 3.38 (3H, s, OMe); δ_C (125 MHz; $CDCl_3$) 165.1, 151.7, 142.6, 134.8, 125.3, 121.6, 115.7, 96.6, 69.4, 65.3, 55.2, 52.8; HRMS Found: 308.0746; $C_{12}H_{15}NO_7$ requires $[M+Na]^+$ 308.0741.

Methyl 3-benzyloxy-4-nitrobenzoate[1]

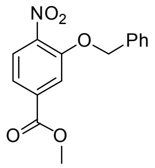

Benzyl bromide (0.41 mL, 3.5 mmol) was added to a suspension of methyl 3-hydroxy-4-nitrobenzoate (0.50 g, 2.5 mmol) and potassium carbonate (1.70 g, 12.5 mmol) in DMF (20 mL) and the reaction heated to 50°C for 16 hours under a drying tube. On completion the reaction was partitioned between ethyl acetate (30 mL) and water (20 mL), the organic layer washed with saturated aqueous sodium bicarbonate solution (2 x 20 mL) and brine (3 x 10 mL), dried over magnesium sulphate and concentrated *in vacuo*. The residue was purified by column chromatography, eluting with methylene chloride, to yield the *title compound* as a colourless solid (0.378 g, 53%). ν_{max}/cm^{-1} (solid state) 3443, 3123, 2968, 2159, 1961, 1689; δ_H (500 MHz; $CDCl_3$) 7.80 (1H, d, *J* 7.8, Ar-H), 7.79 (1H, d, *J* 1.7, Ar-H), 7.66 (1H, dd, *J* 8.2, 1.7, Ar-H), 7.45 (2H, m, Ar-H), 7.37 (2H, m, Ar-H), 7.31 (1H, m, Ar-H), 5.24 (2H, s, OCH_2PH), 3.92 (3H, s, OMe); δ_C (125 MHz; $CDCl_3$) 165.1, 151.40, 142.7, 135.1, 134.8, 128.7, 128.4, 127.1, 125.4, 121.6, 115.9, 71.2, 52.8; HRMS *m/z* (ESI) Found: 310.0685, $[M+Na]^+$ requires 310.0686.

Methyl 3-methoxy-4-nitrobenzoate

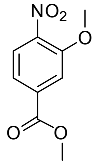

Methyl iodide (1.5 mL, 25 mmol) was added to a suspension of methyl 3-hydroxy-4-nitrobenzoate (1 g, 5 mmol) and potassium carbonate (3.5 g, 25 mmol) in DMF (30 mL) and the reaction heated to 50°C for 1 hour under a drying tube. On completion the reaction was partitioned between ethyl acetate (30 mL) and water (20 mL), the organic layer washed with saturated aqueous sodium bicarbonate solution (2 x 20ml) and brine (3 x 10 mL), dried over magnesium sulphate and concentrated *in vacuo* to yield the *title compound* as a yellow solid (744 mg, 64%). ν_{max}/cm^{-1} (solid state) 3426, 2957, 1727, 1611, 1523; δ_H (500 MHz; $CDCl_3$) 7.78 (1H, d, *J* 8.2, Ar-H), 7.71 (1H, s, Ar-H),

7.63 (1H, d, *J* 8.2, Ar-H), 3.98 (3H, s, OMe), 3.93 (3H, s, OMe); δ_C (125 MHz; $CDCl_3$) 165.1, 152.3, 142.2, 134.2, 125.2, 121.3, 114.5, 56.7, 52.7; HRMS *m/z* (ESI) Found: 234.0371, $[M+Na]^+$ requires 234.0373.

3-Isopropyloxy-4-nitrobenzoic acid[1]

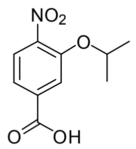

Prepared using procedure A from 3-isopropyloxy-4-nitrobenzoate (100mg, 0.42 mmol) to give pale yellow needles (41.5 mg, 44%); R_f 0.2 (DCM); m.p. 166-170 °C (H_2O); ν_{max}/cm^{-1} (solid state) 2970, 2534, 2159, 1737; δ_H (500 MHz; $CDCl_3$) 7.80 (1H, m, Ar-H), 7.78 (1H, s, Ar-H), 7.74 (1H, m, Ar-H), 4.79 (1H, spt, *J* 6.0, $C_\alpha H$), 1.43 (6H, d, *J* 6.4, $C_\beta H_3$ and $C_\gamma H_3$); δC (125 MHz; $CDCl_3$) 170.1, 150.8, 144.4, 133.2, 125.1, 121.8, 117.3, 73.1, 21.7; HRMS Found: 225.0622; $C_{10}H_{11}NO_5$ requires $[M+H]^+$ 225.0637

3-(2-Methyl-propyloxy)-4-nitrobenzoic acid[2]

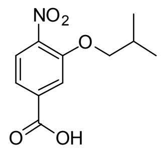

Prepared using procedure A from methyl 3-(2-methyl-propyloxy)-4-nitrobenzoate (400 mg, 1.58 mmol) to give pale yellow needles (345 mg, 73%); R_f 0.2 (DCM); m.p. 188-191 °C (MeOH); ν_{max}/cm^{-1} (solid state) 2962 (broad), 1715, 1610; δ_H (500 MHz; $CDCl_3$) 7.87 (1H, d, *J* 8.3, ArH), 7.81 (1H, d, *J* 1.4, ArH), 7.78 (1H, dd, *J* 8.3, 1.4, ArH), 3.97 (2H, d, *J* 6.4, $C_\alpha H_2$), 2.20 (1H, sept, *J* 6.4, $C_\beta H$), 1.10 (6H, d, *J* 6.7, $C_\gamma H_3$ and $C_\delta H_3$); δ_C (125 MHz; $CDCl_3$) 169.6, 152.1, 143.2, 133.6, 125.3, 121.8, 115.8, 91.9, 28.2, 19.0; HRMS Found: 239.0805; $C_{12}H_{15}NO_5$ requires $[M+Na]^+$ 239.0794.

3-[2-(Methoxymethoxy)ethoxy]-4-nitrobenzoic acid[2]

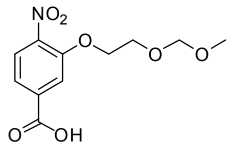

Prepared using procedure A from methyl 3-[2-(methoxymethoxy)ethoxy]-4-nitrobenzoate (400 mg, 1.4 mmol) to give a colourless solid, (262.6 mg, 69%), m.p. 131-133 °C; ν_{max}/cm^{-1} (film) 3063, 2938, 2542, 2159, 2025, 1691; δ_H (500 MHz; $CDCl_3$) 7.90-7.87 (2H, m, Ar-H), 7.81 (1H, dd, *J* 8.2, 1.4, Ar-H), 4.76 (2H, s, OCH_2O), 4.41 (2H, t, *J* 4.4, CH_2), 4.00 (2H, t, *J* 4.4, CH_2), 3.44 (3H, s, OMe); δ_C (125 MHz; $CDCl_3$) 169.4, 151.7, 143.3, 133.8, 125.4, 122.4, 116.3, 96.6, 69.5, 65.4, 55.3; HRMS Found: 294.0571, $C_{11}H_{13}NO_7$ requires $[M+Na]^+$ 294.0584;

3-methoxy-4-nitrobenzoic acid

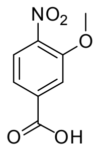

Methyl 3-methoxy-4-nitrobenzoate (500 mg, 2.37 mmol) was dissolved in methanol (10 mL) and 2M aqueous sodium hydroxide (3 ml) added to the stirred solution. After 30 minutes volatiles were removed *in vacuo* and the remaining solution acidified with 2M aqueous HCl. The resulting precipitate was collected by filtration and dried in vacuo to yield the *title compound* as a colourless solid (458 mg, quant.). ν_{max}/cm^{-1} (solid state) 3006, 2543, 1689, 1611; δ_H (500 MHz; d_6-DMSO) 7.97 (1H, d, *J* 8.2, Ar-H), 7.76 (1H, d, *J* 1.4, Ar-H), 7.64 (1H, dd, *J* 8.2, 1.4, Ar-H), 3.99 (3H, s, OMe); δ_C (125 MHz; d_6-DMSO) 165.7, 151.4, 141.9, 135.7, 124.9, 121.2, 114.5, 56.7; HRMS *m/z* (ESI) Found: 196.024756, [M-H]$^-$ requires 196.025146;

Methyl 3-isopropyloxy-4-aminobenzoate[1]

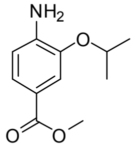

Prepared using procedure B from methyl 3-isopropyloxy-4-nitrobenzoate (100 mg, 0.42 mmol) and purified by flash column chromatography eluting with DCM to give a pale orange oil (72 mg, 77%); ν_{max}/cm^{-1} (solid state) 3488, 3371, 2978, 1703; δ_H (500 MHz, $CDCl_3$) 7.55 (dd, *J* 8.15, 1.8 Hz, 1 H, Ar-H), 7.49 (d, *J* 1.6, 1 H, Ar-H), 6.69 (d, *J* 7.9, 1 H, Ar-H), 4.66 (spt, *J* 6.0, 1 H, C_αH), 1.39 (d, *J* 5.9, 6 H, $C_\beta H_3$ and $C_\gamma H_3$); δ_C (125 MHz, $CDCl_3$) 167.6, 144.1, 142.2, 123.8, 119.4, 114.0, 113.4, 70.8, 51.6, 22.1; HRMS Found: 210.1122; $C_{11}H_{16}NO_3$ requires [M+H]$^+$ 210.1125.

Methyl 3-(2-methyl-propyloxy)-4-amino-benzoate[2]

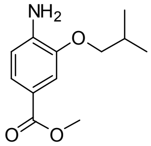

Prepared using procedure B from methyl 3-(2-methyl-propyloxy)-4-nitrobenzoate (200 mg, 0.79 mmol) to give a pale yellow oil which crystallized on standing (125 mg, 71%). R_f 0.5 (DCM); m.p. 61-62 °C (from DCM); ν_{max}/cm^{-1} (solid state) 3462, 3341, 3202, 2951; δ_H (500 MHZ; $CDCl_3$) 7.45 (1H, dd, *J* 8.2, 1.8, Ar-H) 7.35 (1H, d, *J* 1.7, Ar-H), 6.58 (1H, d, *J* 8.2, Ar-H), 4.18 (2H, s, broad, NH_2), 3.77 (3H, s, OCH_3), 3.73 (2H, d, *J* 6.5, $C_\alpha H_2$), 2.04 (1H, sept, *J* 6.7, C_βH), 0.96 (6H, d, *J* 6.7, $C_\gamma H_3$ and $C_\delta H_3$); δ_C (125 MHz, $CDCl_3$) 167.4, 145.6, 141.4, 123.9, 119.3, 113.1, 74.7, 51.6, 28.3, 19.3; HRMS Found: 223.1212; $C_{11}H_{16}NO_3$ requires [M+H]$^+$ 223.1208.

OLIGOMER SYNTHESIS

O_2N-[*O*-^{i}Bu(3-HABA)]-[*O*-^{i}Pr (3-HABA)]-COOMe

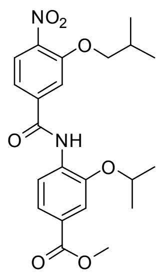

Prepared using procedure D on a 0.34 mmol scale to give a pale yellow oil (94 mg, 46%); R_f 0.25 (DCM); ν_{max}/cm^{-1} (film) 3421, 2979, 2504, 2159, 2030, 1976, 1717; δ_H (500 MHz; $CDCl_3$) 8.80 (1H, s, Amide NH), 8.59 (1H, d, *J* 8.2, Ar-H), 7.93 (1H, d, *J* 8.2, Ar-H), 7.74 (1H, dd, *J* 8.5, 1.6, Ar-H), 7.68 (1H, d, *J* 1.4, Ar-H), 7.61 (1H, d, *J* 1.8, Ar-H), 7.37 (1H, dd, *J* 8.2, 1.4, Ar-H), 4.78 (1H, spt, *J* 6.1, 2-C_αH), 3.97 (2H, d, *J* 6.4, 1-$C_\alpha H_2$), 3.32 (3H, s, OMe), 2.19 (1H, spt, *J* 6.6, 2-C_αH), 1.44 (6H, d, *J* 6, 2-$C_\beta H_3$ and 1-$C_\gamma H_3$), 1.07 (6H, d, *J* 6.4, 1-$C_\gamma H_3$ and 1-$C_\delta H_3$); δ_C (125 MHz; $CDCl_3$) 166.6, 163.0, 152.7, 145.6, 141.6, 139.7, 132.1, 125.8, 123.2, 118.8, 117.2, 114.9, 114.0, 113.1, 76.1, 71.8, 52.1, 28.1, 21.8, 18.9; HRMS Found 431.1816, $C_{22}H_{26}N_2O_7$ $[M+H]^+$ requires 431.1813.

O_2N-[*O*-^{i}Bu(3-HABA)]-[*O*-^{i}Bu (3-HABA)]-COOMe

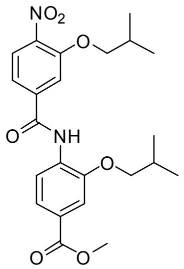

Prepared using procedure D on a 0.42 mmol scale to give pale yellow needles (92.3 mg, 49%); R_f 0.2 (DCM); m.p. 146-149 °C (DCM); ν_{max}/cm^{-1} (solid state) 3431, 3085, 2971, 1722; δ_H (500 MHz; $CDCl_3$) 8.70 (1H, s, Amide N-H), 8.50 (1H, d, *J* 8.7, Ar-H), 7.83 (1H, d, *J* 7.9, Ar-H), 7.66 (1H, d, *J* 8.3, Ar-H), 7.56 (1H, s, Ar-H), 7.50 (1H, s, Ar-H), 7.31 (1H, d, J 7.9, Ar-H), 3.87 (4H, m, 1-$C_\alpha H_2$ and 2- $C\alpha H_2$), 3.84 (3H, s, OMe), 2.12 (2H, m, 1-C_βH and 2-C_βH), 1.03 (6H, d, *J* 6.8, 1-$C_\gamma H_3$ and 1-$C_\delta H_3$), 0.99 (6H, d, *J* 6.8, 2-$C_\gamma H_3$ and 2-$C_\delta H_3$); δ_C (125 MHz; $CDCl_3$) 166.5, 163.0, 152.8, 147.1, 141.6, 139.7, 131.4, 125.9, 125.8, 123.3, 118.6, 117.3, 113.7, 111.6, 76.1, 75.1, 52.1, 28.3, 28.2, 19.3, 19.0; HRMS Found 467.1789, $C_{23}H_{28}N_2O_7$ $[M+Na]^+$ requires 467.1789.

H_2N-[*O*-^{i}Bu(3-HABA)]-[*O*-^{i}Pr(3-HABA)]-COOMe

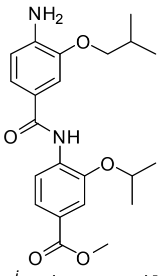

Prepared using procedure B from O_2N-[*O*-^{i}Bu(3-HABA)]-[*O*-^{i}Pr (3-HABA)]-COOMe (94 mg, 0.22 mmol) to give a colourless oil (47 mg, 53%); R_f 0.3 (2% Et_2O in DCM); ν_{max}/cm^{-1} (film) 3490, 3430, 3370, 2959, 1713; δ_H (500 MHz; $CDCl_3$) 8.79 (1H, s, Amide NH), 8.65 (1H, d, *J* 8.2, Ar-H), 7.74 (1H, dd, *J* 8.7, 1.4, Ar-H), 7.61 (1H, s, Ar-H), 7.45 (1H, d, *J* 1.8, Ar-H), 7.31 (1H, d, *J* 1.4, Ar-H), 6.76 (1H, d, *J* 8.2, Ar-H), 4.78 (1H, spt, *J* 6, 2-C_αH), 4.27 (2H, s, NH_2), 3.93 (3H, s, OMe), 3.89 (2H, d, *J* 6.4, 1-$C_\alpha H_2$), 2.19 (1H, spt, *J* 6.6, 1-C_βH), 1.46 (6H, d, *J* 6, 2-$C_\beta H_3$ and 2-$C_\gamma H_3$),1.09 (6H, d, *J* 6.9, 1-$C_\gamma H_3$ and 1-$C_\delta H_3$); δ_C (125 MHz; $CDCl_3$) 166.9, 165.0, 146.2, 145.5, 140.5, 133.5, 124.4, 124.1, 123.4, 119.8, 118.4, 113.3, 113.1, 111.5, 74.7, 71.7, 52.0, 28.2, 19.3, 19.2; HRMS Found 423.1897, $C_{22}H_{28}N_2O_5$ $[M+Na]^+$ requires 423.1890.

H_2N-[*O*-^{i}Bu(3-HABA)]-[*O*-^{i}Bu (3-HABA)]-COOMe

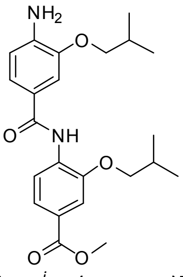

Prepared using procedure B from O_2N-[*O*-^{i}Bu(3-HABA)]-[*O*-^{i}Bu (3-HABA)]-COOMe (63 mg, 0.14 mmol) to give off-white needles (41.4 mg, 71%); R_f 0.3 (10% MeOH in DCM); m.p. 147-148 °C (DCM); ν_{max}/cm^{-1} (solid state) 3461, 3435, 3335, 2961, 2529, 2159, 2028; δ_H (500 MHz; $CDCl_3$) 8.73 (1H, s, Amide NH), 8.63 (1H, d, *J* 8.7, Ar-H), 7.72 (1H, dd, *J* 8.8, 1.6, Ar-H), 7.55 (1H, d, *J* 1.8, Ar-H), 7.40 (1H, d, *J* 1.8, Ar-H), 7.30 (1H, dd, *J* 8.2, 1.8, Ar-H), 6.73 (1H, d, *J* 7.8, Ar-H), 4.23 (2H, s (broad), NH_2), 3.92 (2H, d, *J* 6.4, 2-$C_\alpha H_2$), 3.91 (3H, s, OMe), 3.85 (2H, d, *J* 6.4, 2-$C_\alpha H_2$), 2.18 (2H, m, 1-C_βH and 2-C_βH), 1.11 (6H, d, *J* 6.9, 1-$C_\gamma H_3$ and 1-$C_\delta H_3$), 1.06 (6H, d, *J* 6.9, 2-$C_\gamma H_3$ and 2-$C_\delta H_3$); δ_C (125 MHz; $CDCl_3$) 166.9, 165.1, 146.8, 146.2, 140.5, 132.8, 124.4, 124.1, 123.5, 120.1, 118.2, 113.4, 111.4, 110.3, 75.0, 74.7, 52.0, 28.3, 28.3, 19.4, 19.3; HRMS Found: 437.2042; $C_{23}H_{30}N_2O_5$ $[M+Na]^+$ requires 437.2047.

O_2N-[*O*-^{i}Bu(3-HABA)]-[*O*-^{i}Bu (3-HABA)]-[*O*-^{i}Pr(3-HABA)]-COOMe

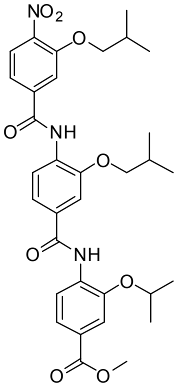

Prepared using procedure D on a 0.11 mmol scale to give a yellow solid (21.3 mg, 30%); R_f 0.25 (2% Et_2O in DCM); m.p. 189-191 °C (DCM); ν_{max}/cm^{-1} (solid state) 2966, 2514, 2159, 2026, 1706, 1680; δ_H (500 MHz; $CDCl_3$) 8.90 (1H, s, Amide NH), 8.81 (1H, s, Amide NH), 8.69 (1H, d, *J* 8.2, Ar-H), 8.65 (1H, d, *J* 8.7, Ar-H), 7.96 (1H, d, *J* 8.2, Ar-H), 7.76 (1H, dd, *J* 8.7, 1.4, Ar-H), 7.69 (1H, s, Ar-H), 7.64 (2H, d, *J* 6.9, Ar-H), 7.46 (2H, dd, *J* 12.1, 8.5, Ar-H), 4.81 (1H, spt, *J* 6, 3-C_αH), 4.01 (4H, dd, *J* 10.5, 6.4, 1-$C_\alpha H_2$ and 2-$C_\alpha H_2$), 3.94 (3H, s, OMe), 2.24 (2H, m, 1-C_βH and 2-C_βH), 1.49 (6H, d, *J* 6.4, 3-$C_\gamma H_3$ and 3-$C_\delta H_3$), 1.15 (6H, d, *J* 6.9, 1-$C_\gamma H_3$ and 1-$C_\delta H_3$), 1.11 (6H, d, *J* 6.9, 2-$C_\gamma H_3$ and 2-$C_\delta H_3$); δ_C (125 MHz; $CDCl_3$) 166.7, 164.2, 163.1, 152.8, 147.8, 145.8, 141.7, 139.6, 132.9, 130.7, 130.6, 125.9, 125.1, 123.3, 118.9, 118.9, 118.6, 117.4, 113.7, 113.1, 110.6, 76.1, 75.2, 71.8, 52.1, 28.2, 28.2, 22.2, 19.3, 19.0; HRMS Found: 644.2596; $C_{33}H_{39}N_3O_9$ $[M+Na]^+$ requires 644.2579.

O_2N-[*O*-^{i}Pr(3-HABA)]-[*O*-^{i}Bu (3-HABA)]-[*O*-^{i}Bu(3-HABA)]-COOMe

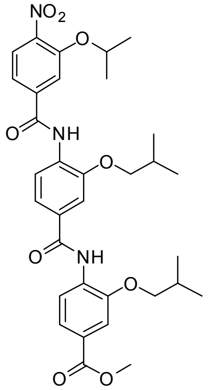

Prepared using procedure D on a 0.1 mmol scale and further purified by trituration with hexane to give pale yellow needles (5.5 mg, 9%); R_f 0.3 (99:1 DCM–Et_2O); m.p. 195-196 °C (Hexane); ν_{max}/cm^{-1} (solid state) 3437, 2961, 2874, 1709, 1680; δ_H (500 MHz; $CDCl_3$) 8.88 (1H, s, Amide NH), 8.78 (1H, s, Amide NH), 8.66 (1H, d, *J* 8.2, Ar-H), 8.63 (1H, d, *J* 8.2, Ar-H), 7.88 (1H, s, Ar-H), 7.87 (1H, s, Ar-H), 7.75 (1H, dd, *J* 8.5, 1.1, Ar-H), 7.70 (1H, s, Ar-H), 7.58 (1H, s, Ar-H), 7.46 (1H, d, *J* 7.8, Ar-H), 7.38 (1H, d, *J* 8.2, Ar-H), 4.82 (1H, spt, *J* 6, 1-C_αH), 3.98 (2H, d, *J* 6.9, 1-$C_\alpha H_2$), 3.95 (2H, d, *J* 6.4, 2-$C_\alpha H_2$), 3.92

(3H, s, OMe), 2.23 (2H, m, 1-C_βH and 2-C_βH), 1.45 (6H, d, *J* 6, 3-$C_\beta H_3$ and 3-$C_\gamma H_3$), 1.13 (6H, d, *J* 6.9, 1-$C_\gamma H_3$ and 1-$C_\delta H_3$), 1.11 (6H, d, *J* 6.9, 2-$C_\gamma H_3$ and 2-$C_\delta H_3$); δ_C (125 MHz; $CDCl_3$) 166.7, 164.3, 163.2, 151.6, 147.8, 147.0, 143.2, 143.0, 139.3, 132.1, 130.8, 130.6, 125.8, 125.1, 123.4, 118.9, 118.4, 117.1, 115.4, 111.5, 110.5, 75.2, 75.1, 73.2, 52.1, 28.3, 28.2, 21.8, 19.4, 19.3; HRMS Found: 644.2567; $C_{33}H_{39}N_3O_9$ $[M+Na]^+$ requires 644.2579.

H_2N-[*O*-^{i}Bu(3-HABA)]-[*O*-^{i}Bu (3-HABA)]-[*O*-^{i}Pr(3-HABA)]-COOMe

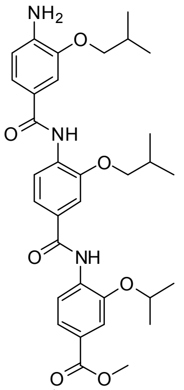

Prepared using procedure B from O_2N-[O-^{i}Bu(3-HABA)]-[O-^{i}Bu (3-HABA)]-[O-^{i}Pr(3-HABA)]-COOMe (15 mg, 24 µmol) to give a colourless solid (4.4 mg, 31%). m.p. 197-198 °C (CH_2Cl_2); R_f 0.1 (2:98 Et_2O–CH_2Cl_2); ν_{max}/cm^{-1} (solid state) 3491, 3436, 3350, 2960, 1714; δ_H (500 MHz; $CDCl_3$) 8.88 (1H, s, Amide NH), 8.73 (1H, s, Amide NH), 8.70 (1H, d, *J* 8.5, Ar-H), 8.63 (1H, d, *J* 8.5, Ar-H), 7.73 (1H, dd, *J* 8.5, 1.7, Ar-H), 7.60 (2H, dd, *J* 4.7, 1.7, Ar-H), 7.42 (2H, m, Ar-H), 7.33 (1H, dd, *J* 8.1, 1.7, Ar-H), 6.75 (1H, d, *J* 8.1, Ar-H), 4.77 (1H, spt, *J* 6.1, 3-$C_\alpha H_2$), 4.23 (2H, br. s, NH_2), 3.98 (2H, d, *J* 6.4, 1-$C_\alpha H_2$), 3.92 (3H, s, OCH_3), 3.87 (2H, d, *J* 6.8, 2-$C_\alpha H_2$), 2.23 (1H, spt, *J* 6.4, 1-C_βH), 2.18 (1H, spt, J 6.8, 2-C_βH), 1.46 (6H, d, *J* 6.0, 3-$C_\beta H_3$ and 3-$C_\gamma H_3$), 1.13 (6H, d, *J* 6.8, 1-$C_\gamma H_3$ and 1-$C_\delta H_3$), 1.08 (6H, d, *J* 6.4, 2-$C_\gamma H_3$ and 2-$C_\delta H_3$); δ_C (125 MHz; $CDCl_3$) 166.8, 165.1, 164.6, 147.6, 146.2, 145.7, 140.6, 133.2, 132.0, 129.1, 124.9, 124.0, 123.3, 120.1, 119.0, 118.5, 118.4, 113.4, 113.1, 110.4, 110.3, 75.1, 74.8, 71.9, 52.0, 28.4, 28.3, 22.2, 19.4, 19.3; HRMS Found: 614.2825; $C_{33}H_{41}N_3O_7$ requires $[M+Na]^+$ 614.2837.

H_2N-[*O*-^{i}Pr(3-HABA)]-[*O*-^{i}Bu (3-HABA)]-[*O*-^{i}Bu(3-HABA)]-COOMe

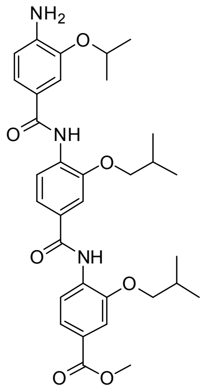

Prepared using procedure B from O_2N-[*O*-^{i}Pr(3-HABA)]-[*O*-^{i}Bu (3-HABA)]-[*O*-^{i}Bu(3-HABA)]-COOMe (89 mg, 0.14 mmol) to give a colourless solid (34 mg, 41%). m.p. 210-212 °C (CH_2Cl_2); R_f 0.25 (1:99 MeOH–CH_2Cl_2); ν_{max}/cm^{-1} (solid state) 3496, 3443, 3376, 2962, 2893, 1697, 1673; δ_H (500 MHz; $CDCl_3$) 8.90 (1H, s, Amide NH), 8.77 (1H, s, Amide NH), 8.73 (1H, d, *J* 8.7, Ar-H), 8.67 (1H, d, *J* 8.7, Ar-H), 7.77 (1H, d, *J* 8.7, Ar-H), 7.61 (2H, dd, *J* 4.6, 1.4, Ar-H), 7.49 (1H, d, *J* 1.4, Ar-H), 7.46 (1H, dd, *J* 8.5, 1.4, Ar-H), 7.32 (2H, dd, *J* 8.5, 1.4, Ar-H), 6.80 (1H, d, *J* 8.2, Ar-H), 4.72 (1H, spt, *J* 6, 1-C_αH), 3.99 (2H, d, *J* 6.4, 2-$C_\alpha H_2$), 3.97 (2H, d, *J* 6.4, 3-$C_\alpha H_2$), 3.95 (3H, s, CO_2Me), 2.25 (2H, m, 2-C_βH and 3-C_βH), 1.43 (6H, d, *J* 6, 1-$C_\beta H_3$ and 1-$C_\gamma H_3$), 1.15 (12H, overlapping doublets, *J* 6, 2-$C_\gamma H_3$, 2-$C_\delta H_3$, 3-$C_\gamma H_3$ and 3$C_\delta H_3$); δ_C (125 MHz; $CDCl_3$) 166.8, 165.1, 164.6, 147.6, 147.0, 145.0, 132.4, 132.0, 129.1, 124.9, 123.4, 119.9, 119.0, 118.5, 118.3, 113.9, 112.3, 111.5, 110.2, 75.1, 70.9, 52.0, 28.4, 28.3, 22.2, 19.4, 19.3; HRMS Found: 614.2834; $C_{33}H_{41}N_3O_7$ requires $[M+Na]^+$ 614.2837.

H_2N-[*O*-^{i}Bu(3-HABA)]-[*O*-^{i}Bu (3-HABA)]-[*O*-^{i}Pr(3-HABA)]-COOH

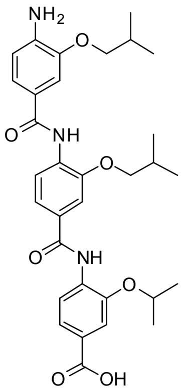

3

Prepared using procedure A from H_2N-[*O*-^{i}Bu(3-HABA)]-[*O*-^{i}Bu (3-HABA)]-[*O*-^{i}Pr(3-HABA)]-COOMe (43 mg, 0.07 mmol) to give the product as a pale yellow solid (18.4 mg, 45%); m.p. 211-213 °C (CH_2Cl_2–MeOH); ν_{max}/cm^{-1} (solid state) 3365, 3480, 2958, 2874, 2552, 1682; δ_H (500 MHz; d_6-DMSO) 9.33 (1H, s, NH), 9.10 (1H, s, NH), 8.25 (1H, d, *J* 8.5, Ar-H), 8.19 (1H, d, *J* 8.1, Ar-H), 7.61 (1H, dd, *J* 8.1, 1.7, Ar-H), 7.58 (3H, m, Ar-H), 7.41 (1H, dd, *J* 8.1, 2.1, Ar-H), 7.38 (1H, d, *J* 1.7, Ar-H), 6.86 (1H, d, *J*

8.1, Ar-H), 4.74 (1H, spt, *J* 6.1, 3-C_αH), 3.97 (2H, d, *J* 6.4, 1-$C_\alpha H_2$), 3.83 (2H, d, *J* 6.8, 2-$C_\alpha H_2$), 2.17 (1H, spt, *J* 6.4, 1-C_βH), 2.11 (1H, spt, *J* 6.8, 2-C_βH), 1.37 (6H, d, *J* 6.1, 3-$C_\beta H_3$ and 3-$C_\gamma H_3$), 1.06 (6H, d, *J* 6.4, 1-$C_\gamma H_3$ and 1-$C_\delta H_3$), 1.04 (6H, d, *J* 6.8, 2-$C_\gamma H_3$ and 1-$C_\delta H_3$); δ_C (125 MHz; d_6-DMSO) 166.8, 164.5, 164.4, 148.5, 148.4, 147.4, 144.8, 132.5, 131.3, 129.2, 126.6, 126.5, 122.2, 121.3, 120.4, 120.0, 119.9, 113.9, 112.5, 110.5, 109.8, 74.5, 74.0, 71.4, 27.8, 28.7, 21.6, 19.1, 19.0; HRMS Found: 600.2673; $C_{32}H_{39}N_3O_7$ requires $[M+Na]^+$ 600.2680.

H_2N-[*O*-^{i}Pr(3-HABA)]-[*O*-^{i}Bu (3-HABA)]-[*O*-^{i}Bu(3-HABA)]-COOH

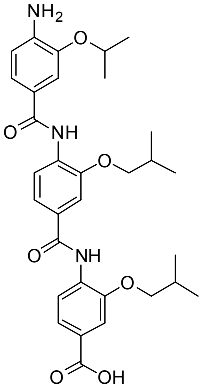

4

Prepared using procedure A from H_2N-[*O*-^{i}Pr(3-HABA)]-[*O*-^{i}Bu (3-HABA)]-[*O*-^{i}Bu(3-HABA)]-COOMe (24 mg, 0.04 mmol) to give the product as a pale yellow solid (20 mg, 86%). m.p. 209-211 °C (CH_2Cl_2); ν_{max}/cm^{-1} (solid state) 3379, 2959, 2927, 2549, 1715; δ_H (500 MHz; d_6-DMSO) 9.42 (1H, s, NH), 9.16 (1H, s, NH), 8.15 (1H, d, *J* 8.1, Ar-H), 8.08 (1H, d, *J* 8.5, Ar-H), 7.60 (2H, m, Ar-H), 7.57 (1H, dd, *J* 8.5, 1.4, Ar-H), 7.55 (1H, d, *J* 1.7, Ar-H), 7.44 (1H, d, *J* 1.4, Ar-H), 7.39 (1H, dd, *J* 8.3, 1.9, Ar-H), 6.92 (1H, d, *J* 7.7, Ar-H), 4.64 (1H, spt, *J* 6.1, 1-C_αH), 3.93 (2H, d, *J* 6.4, 2-$C_\alpha H_2$), 3.90 (2H, d, *J* 6.4, 3-$C_\alpha H_2$), 2.13 (1H, spt, *J* 6.4, 2-$C_\beta H_2$), 2.11 (1H, spt, *J* 6.4, 3-$C_\beta H_2$), 1.31 (6H, d, *J* 6.1, 1-$C_\beta H_3$ and 1-$C_\gamma H_3$), 1.02 (6H, d, *J* 6.4, 2-$C_\gamma H_3$ and 2-$C_\delta H_3$), 1.01 (6H, d, *J* 6.4, 3-$C_\gamma H_3$ and 3-$C_\delta H_3$); δ_C (125 MHz; d_6-DMSO) 166.8, 164.4, 164.2, 149.4, 149.0, 147.2, 143.9, 132.2, 131.5, 129.1, 127.0, 126.8, 122.1, 121.8, 121.3, 121.1, 121.0, 120.0, 112.7, 112.2, 110.6, 110.5, 74.5, 74.4, 70.6, 27.8, 27.7, 21.8, 19.1, 19.0; HRMS Found: 600.2675; $C_{32}H_{39}N_3O_7$ requires $[M+Na]^+$ 600.2680.

LIBRARY SYNTHESIS

The compound library was prepared in parallel using the methods and building blocks described above, checking at pertinent times during the synthesis by crude NMR and LC-MS, to afford the below compounds. Compounds were either pure following final precipitation or purified by preparative HPLC. Where the amount of material produced allowed ^{13}C-NMR spectra were collected otherwise ^{1}H-NMR and high resolution mass spectrometry were used to characterise the compound library.

H_2N-[*O*-Me(3-HABA)]-[*O*-^{i}Pr (3-HABA)]-[*O*-^{i}Bu(3-HABA)]-COOH

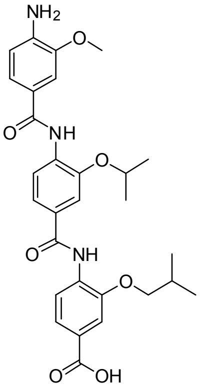

5

δ_H (500 MHz; d_6-DMSO) 9.35 (1H, s, Amide N-H), 8.97 (1H, s, Amide N-H), 8.28 (1H, d, *J* 8.5, Ar-H), 8.07 (1H, d, *J* 8.5, Ar-H), 7.59 (4H, m, Ar-H), 7.35 (2H, m, Ar-H), 6.70 (1H, d, *J* 8.5, Ar-H), 4.78 (1H, spt, *J* 6, 2-C_αH), 3.90 (2H, d, *J* 6.4, 3-$C_\beta H_2$), 3.85 (3H, s, 1-$C_\alpha H_3$), 2.10 (1H, spt, *J* 6.6, 3-C_αH), 1.38 (6H, d, *J* 6, 2-$C_\beta H_3$ and 2-$C_\delta H_3$), 1.02 (6H, d, *J* 6.8, 3-$C_\delta H_3$ and 3$C_\gamma H_3$); HRMS Found: 536.2399, M+H requires 536.2391;

H_2N-[*O*-^{i}Bu(3-HABA)]-[*O*-^{i}Bu (3-HABA)]-[*O*-^{i}Bu(3-HABA)]-COOH

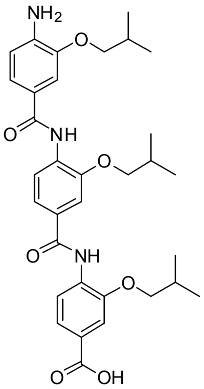

6

δ_H (500 MHz; d_6-DMSO) 9.31 (1H, s, Amide N-H), 9.00 (1H, s, Amide N-H), 8.15 (1H, d, *J* 8.1, Ar-H), 8.00 (1H, d, *J* 8.1, Ar-H), 7.61 (4H, m, Ar-H), 7.35 (2H, m, Ar-H), 6.71 (1H, d, *J* 8.5, Ar-H), 4.05 (2H, d, *J* 6.4, 1-$C_\alpha H_2$), 3.92 (4H, m, 2-$C_\alpha H_2$ and 3-$C_\alpha H_2$), 2.11 (3H, m, 1-C_βH, 2-C_βH and 3-C_βH), 1.02 (18H, m, 1-$C_\gamma H_3$ and $C_\delta H_3$, 2-$C_\gamma H_3$ and $C_\delta H_3$ and 3-$C_\gamma H_3$ and $C_\delta H_3$); HRMS Found: 614.284, M+Na requires 614.2836;

H_2N-[*O*-^{i}Bu(3-HABA)]-[*O*-^{i}Pr (3-HABA)]-[*O*-^{i}Bu(3-HABA)]-COOH

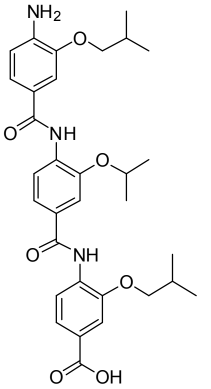

7

δ_H (500 MHz; d_6-DMSO) 9.35 (1H, s, Amide N-H), 8.96 (1H, s, Amide N-H), 8.28 (1H, d, *J* 8.5, Ar-H), 8.07 (1H, d, *J* 8.1, Ar-H), 7.58 (4H, m, Ar-H), 7.32 (2H, m, Ar-H), 6.72 (1H, d, *J* 8.1, Ar-H), 4.78 (1H, spt, *J* 6, 2-$C_\alpha H_2$), 3.89 (2H, d, *J* 6.8, 1-$C_\alpha H_2$), 3.81 (2H, d, *J* 6.8, 3-C_αH), 2.09 (2H, m, 1-C_βH and 3-C_βH), 1.38 (6H, d, *J* 6, 2-$C_\beta H_3$ and 2-$C_\gamma H_3$), 1.03 (6H, d, *J* 6.8, 1-$C_\gamma H_3$ and 1-$C_\delta H_3$), 1.01 (6H, d, *J* 6.8, 3-$C_\gamma H_3$ and 3-$C_\delta H_3$); HRMS Found: 578.2863, M+H requires 578.286;

H_2N-[*O*-Bn(3-HABA)]-[*O*-Bn (3-HABA)]-[*O*-Bn(3-HABA)]-COOH

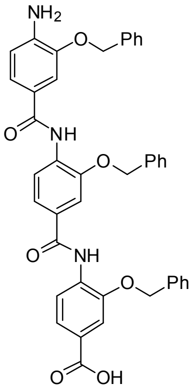

8

δ_H (500 MHz; d_6-DMSO) 9.54 (1H, m, Amide N-H), 9.14 (1H, s, Amide N-H), 8.11 (2H, m, Ar-H), 7.63 (10H, m, Ar-H), 7.48 (2H, m, Ar-H), 7.34 (12H, m, Ar-H), 6.76 (1H, d, *J* 8.1, Ar-H), 5.29 (2H, m, Benzylic CH_2), 5.25 (2H, s, Benzylic CH_2), 5.12 (2H, s, Benzylic CH_2); δ_C (125 MHz; d_6-DMSO) 166.8, 165.8, 164.5, 164.4, 149.3, 149.0, 145.9, 136.6, 136.5, 132.0, 131.6, 131.2, 130.0, 129.9, 128.4, 128.4, 128.0, 127.9, 127.9, 127.8, 127.5, 127.4, 127.3, 127.2, 125.9, 122.5, 121.8, 121.3, 120.3, 115.4, 113.1, 112.9, 111.6, 111.3, 70.3, 70.2, 70.1; HRMS *m/z* (ESI) Found: 716.2352, $[M+Na]^+$ requires 716.2367.

H_2N-[*O*-^{i}Pr(3-HABA)]-[*O*-^{i}Pr (3-HABA)]-[*O*-^{i}Pr(3-HABA)]-COOH

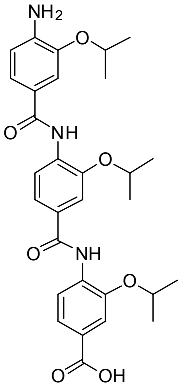

9

δ_H (500 MHz; d_6-DMSO) 9.28 (1H, s, Amide N-H), 8.94 (1H, s, Amide N-H), 8.30 (1H, d, *J* 8.5, Ar-H), 8.18 (1H, d, *J* 8.5, Ar-H), 7.60-7.54 (4H, m, Ar-H), 7.35-7.32 (2H, m, Ar-H), 6.72 (1H, d, *J* 8.1, Ar-H), 4.79 (1H, spt, *J* 6, $C_\alpha H$), 4.72 (1H, spt, *J* 6, $C_\alpha H$), 4.60 (1H, spt, *J* 6, $C_\alpha H$), 1.39 (6H, d, *J* 6, $C_\beta H_3$ and $C_\gamma H_3$), 1.35 (6H, d, *J* 6, $C_\beta H_3$ and $C_\gamma H_3$), 1.32 (6H, d, *J* 6, $C_\beta H_3$ and $C_\gamma H_3$); HRMS Found: 550.2558, M+H requires 550.2547.

H_2N-[*O*-^{i}Bu(3-HABA)]-[*O*-^{i}Pr (3-HABA)]-[*O*-^{i}Pr(3-HABA)]-COOH

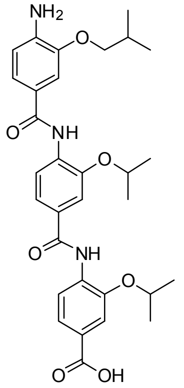

10

δ_H (500 MHz; d_6-DMSO) 9.30 (1H, s, Amide N-H), 9.08 (1H, s, Amide N-H), 8.25 (1H, d, J 8.5, Ar-H), 8.17 (1H, d, J 8.1, Ar-H), 7.59 (4H, m, Ar-H), 7.38 (2H, m, Ar-H), 6.91 (1H, d, J 7.7, Ar-H), 4.79 (1H, spt, J 6, 2-$C_\alpha H$), 4.69 (1H, spt, J 6, 3-$C_\alpha H$), 3.84 (2H, d, J 6.8, 1-$C_\beta H_2$), 2.09 (1H, spt, J 6.8, 1-CαH), 1.39 (6H, d, J 6, 2-$C_\beta H_3$ and 2-$C_\delta H_3$), 1.34 (6H, d, J 6, 3-$C_\beta H_3$ and 3-$C_\delta H_3$), 1.02 (6H, d, J 6.8, 1-$C_\delta H_3$ and 1-$C_\gamma H_3$); δ_C (125 MHz; d_6-DMSO) 166.9, 164.3, 164.2, 147.6, 147.2, 132.4, 132.0, 129.5, 126.6, 122.1, 121.3, 120.9, 120.6, 120.0, 113.9, 112.2, 110.4, 74.2, 71.4, 71.4, 27.7, 21.8, 21.7; HRMS Found: 564.2716, M+H requires 564.2704

H_2N-[*O*-Bn(3-HABA)]-[*O*-^{i}Pr (3-HABA)]-[*O*-^{i}Pr(3-HABA)]-COOH

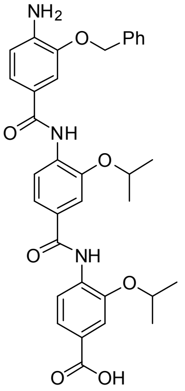

11

δ$_H$ (500 MHz; d_6-DMSO) 9.31 (1H, s, Amide N-H), 9.07 (1H, s, Amide N-H), 8.23 (1H, d, *J* 8.1, Ar-H), 8.16 (1H, d, *J* 8.1, Ar-H), 7.60-7.51 (7H, m, Ar-H), 7.41-7.38 (3H, m, Ar-H), 7.34-7.31 (1H, m, Ar-H), 6.92 (1H, d, *J* 8.1, Ar-H), 5.23 (2H, s, 1-Cα), 4.78 (1H, spt, *J* 6.1, 2-Cα), 4.71 (1H, spt, *J* 6.1, 3-Cα), 1.37 (6H, d, *J* 6.1, 2-Cβ and 2-Cγ), 1.34 (6H, d, *J* 6.1, 3-Cβ and 3-Cγ); δ$_C$ (125 MHz; d_6-DMSO) 166.9, 164.3, 164.2, 147.6, 147.3, 136.9, 132.4, 132.0, 129.5, 128.4, 127.7, 127.3, 126.7, 122.1, 121.3, 121.2, 120.8, 119.9, 113.9, 112.3, 111.4, 71.5, 71.4, 69.5, 21.8, 21.7; HRMS Found: 598.2562, M+H requires 598.2547

H_2N-[*O*-2-hydroxyethyl(3-HABA)]-[*O*-^{i}Pr (3-HABA)]-[*O*-^{i}Pr(3-HABA)]-COOH

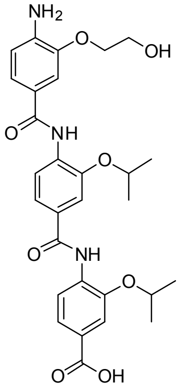

12

δ$_H$ (500 MHz; d_6-DMSO) 9.27 (1H, s, Amide N-H), 8.95 (1H, s, Amide N-H), 8.28 (1H, d, *J* 8.5, Ar-H), 8.16 (1H, d, *J* 8.1, Ar-H), 7.56 (4H, m, Ar-H), 7.35 (2H, m, Ar-H), 6.70 (1H, d, *J* 8.1, Ar-H), 4.79 (1H, spt, *J* 6, 2-Cα), 4.71 (1H, spt, *J* 6, 3-Cα), 4.02 (2H, t, *J* 4.7, 1-Cα), 3.75 (2H, t, *J* 4.7, 1-Cβ), 1.39 (6H, d, *J* 6, 2-Cβ and 2-Cγ), 1.35 (6H, d, *J* 6, 3-Cβ and 3-Cγ); HRMS Found: 552.2343, M+H requires 552.2340;

Compound **13** was synthesised previously[3]

SYNTHESIS OF COMPOUND 2

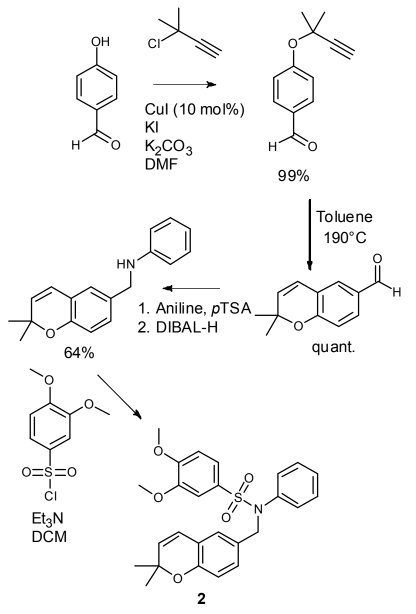

4-(2-methylbut-3-yn-2-yloxy)benzaldehyde

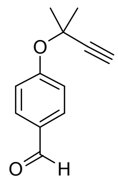

Following a literature procedure,[4] 4-hydroxy benzaldehyde (664 mg, 5.4 mmol), potassium carbonate (1.5 g, 10.8 mmol), potassium iodide (1.5 g, 9.2 mmol) and copper iodide (52 mg, 5 mol%) were dissolved in DMF (20 mL) and 3-chloro-3-methyl-1-butyne (1.1 mL, 9.8 mmol) was added. The reaction was heated to 65 °C for 2.5 hours, allowed to cool to r.t. diluted with ether (30 mL) and washed with 1 M aqueous sodium hydroxide (3 x 15 mL) and brine (2 x 15 mL), dried over magnesium sulphate and concentrated *in vacuo* to yield the *title compound* as an orange oil (1.01 g, 99%). v_{max}/cm^{-1} (solid state) 3291, 2990, 1698, 1601, 1577; δ_H (500 MHz; $CDCl_3$) 9.91 (1H, s, CHO), 7.83 (2H, d, *J* 8.7, Ar-H), 7.35 (2H, d, *J* 8.7, Ar-H), 2.69 (1H, s, Alkynyl CH), 1.73 (6H, s, 2 x Me); δ_C (125 MHz; $CDCl_3$) 190.9, 161.1, 131.3, 131.2, 119.6, 84.8, 73.4, 72.4, 29.4; HRMS *m/z* (ESI) Found: 188.0842, M+H Requires 188.0837.

2,2-dimethyl-2H-chromene-6-carbaldehyde

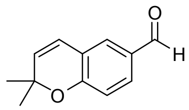

Following a literature proceedure,[4] 4-(2-methylbut-3-yn-2-yloxy)benzaldehyde (1 g, 5.3 mmol) was dissolved in toluene (5 mL) and heated to 190 °C under microwave irradiation for 25 minutes, allowed to cool, diluted with ethyl acetate and concentrated *in vacuo* to yield the *title compound* as

a dark orange oil (1 g, quant.). v_{max}/cm^{-1} (solid state) 2976, 2648, 1690, 1600; δ_H (500 MHz; $CDCl_3$) 9.83 (1H, s, CHO), 7.66 (1H, dd, *J* 8.2, 1.4, Ar-H), 7.52 (1H, dd, *J* 1.4, Ar-H), 6.87 (1H, dd, *J* 8.2, Ar-H), 6.37 (1H, d, *J* 10.1, Alkene), 5.70 (1H, d, *J* 9.6, Alkene), 1.48 (6H, s, 2 x Me); δ_C (125 MHz; $CDCl_3$) 190.9, 158.7, 131.9, 131.4, 129.9, 127.8, 121.2, 116.8, 116.0, 77.9, 28.5; HRMS *m/z* (ESI) Found: 188.0830, M+H Requires 188.0837.

***N*-[(2,2-dimethyl-2H-chromen-6-yl)methyl]aniline**

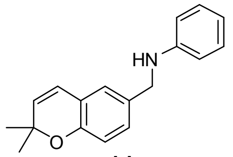

Prepared using a modified literature procedure,[4] aniline (1 mL, 10.6 mmol) was added to a solution of 2,2-dimethyl-2H-chromene-6-carbaldehyde (1 g, 5.3 mmol) and *p*-toluenesulfonic acid (200 mg, 1.06 mmol) in methylene chloride (20 mL) and heated to reflux for 3 hours. The reaction mixture was concentrated *in vacuo* and NMR used to confirm full conversion to imine, then the residue dissolved in toluene (30 mL) and cooled to 0 °C (ice bath). Diisobutylaluminum hydride (1 M in hexanes, 10.6 mL, 10.6 mmol) was added dropwise and the reaction stirred overnight and allowed to warm to r.t., cooled to 0 °C and quenched by the dropwise addition of saturated aqueous ammonium chloride solution with vigorous stirring. The resulting precipitate was removed by filtration and the filtrate diluted with ethyl acetate (30 ml), washed with water (10 mL), 1M aqueous HCl (3 x 10 mL) and brine (10 ml), dried over magnesium sulphate and concentrated *in vacuo* to yield the *title compound* as a brown oil (907 mg, 64%). v_{max}/cm^{-1} (solid state) 3417, 2974, 2864, 1603, 1505; δ_H (500 MHz; $CDCl_3$) 7.09 (2H, t, *J* 7.8, Aniline Ar-H), 7.00 (1H, dd, *J* 8.2, 1.8, Chromene Ar-H), 6.89 (1H, dd, *J* 1.4, Chromene Ar-H), 6.67-6.61 (2H, m, Aniline and Chromene Ar-H), 6.55 (2H, dd, *J* 7.8, Aniline Ar-H), 6.20 (1H, d, *J* 9.6, Alkene), 5.52 (1H, d, *J* 9.6, Alkene), 4.1 (2H, s, Benzylic CH_2), 3.88 (1H, s (br), NH), 1.34 (6H, s, 2 x Me); δ_C (125 MHz; $CDCl_3$) 152.2, 148.2, 131.4, 131.0, 129.2, 128.4, 125.6, 122.2, 121.3, 117.5, 116.4, 112.9, 76.2, 47.9, 28.0; HRMS *m/z* (ESI) *m/z* (ESI) Found: 266.15398, $[M+H]^+$ requires 266.153941.

***N*-[(2,2-dimethyl-2H-chromen-6-yl)methyl]-3,4-dimethoxy-N-phenylbenzene-1-sulfonamide**

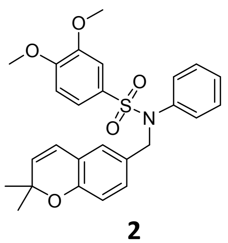

2

Prepared using a modified literature procedure,[5] 3,4-dimethoxybenzene sulfonyl chloride (890 mg, 3.77 mmol) was added to a solution of N-[(2,2-dimethyl-2H-chromen-6-yl)methyl]aniline (500 mg,

1.88 mmol) and triethylamine (1.3 mL, 9.4 mmol) in methylene chloride (50 mL) and the reaction heated to reflux overnight, allowed to cool to r.t. and diluted with methylene chloride, washed with water, saturated aqueous sodium bicarbonate, 1M aqueous HCl, and brine, dried over magnesium sulphate and concentrated *in vacuo*. The residue was purified by column chromatography eluting with 20-30% ethyl acetate in petrol to yield the *title compound* as a yellow solid (596 mg, 68%). ν_{max}/cm^{-1} (solid state) 2974, 2934, 1586, 1507; δ_H (500 MHz; $CDCl_3$) 7.33 (1H, dd, *J* 8.5, 2.1, Ar-H), 7.23-7.19 (3H, m, Ar-H), 7.01-6.99 (2H, m, Ar-H), 6.96 (1H, dd, *J* 1.8, Ar-H), 6.93-6.86 (3H, m, Ar-H), 6.59 (1H, d, *J* 8.2, Ar-H), 6.21 (1H, d, *J* 10.1, Alkene), 5.56 (1H, d, *J* 9.6, Alkene), 4.62 (2H, s, Benzylic CH_2), 3.93 (3H, s, OMe), 3.74 (3H, s, OMe), 1.37 (6H, s, 2 x Me); HRMS *m/z* (ESI) Found: 466.168811, $[M+H]^+$ requires 466.16827.

PROTEIN EXPRESSION AND PURIFICATION

Plasmids

P300 (330-420)

The cDNA coding for human p300 CH1 domain (amino acid residues 330-420) was subcloned into the *BamHI* and *XhoI* site of pGEX-6p-2 (GE healthcare) to generate the plasmid pGEX-p300..

eIF4e (35-206)

The cDNA coding for human eIF4e (amino acid residues 35-206) was subcloned into the *BamHI* and *XhoI* sites of pET-28a (Novagen) with an N-terminal His-SUMO tag to generate the plasmid pETsumo-eIF4e.

Protein Expression

P300 (330 – 420)

pGEX-p300 was transformed in to BL21 (DE3) pLysS Gold cells and incubated at 37°C with aeration until an OD_{600} 0.6-0.8 was reached. Protein expression was induced by addition of 1mM sterile IPTG to the culture and the culture was then incubated at 18°C with aeration overnight in the presence of 50 µM Zinc. Cells were harvested by centrifugation at 3500 × g at 4°C for 20 minutes. Cell pellets were resuspended in Lysis buffer (20 mM TRIS pH 7.9, 500 mM NaCl, 0.1% (v/v) triton X-100). 5 U DNase I and 2 mM $MgCl_2$ were added to the suspensions prior to lysing the cells. Cells were lysed by sonication on ice for 10 cycles of 20 sec on 40 sec off, until the lysate was clear and no more DNA was released. The lysate was centrifuged at 13000 × g for 25 minutes at 4°C. The supernatant was decanted and filtered through a 0.45 µm Amicon syringe filter.

The fusion protein was purified with approximately 10 mL Glutathione Superflow Resin (Generon) packed in a free-flow gravity column. The lysate was then added to the column and mixed with the beads. The mixture was left for 10 min to allow binding. The column was washed with 5 column

volumes of Lysis Buffer, then with 5 column volumes of Lysis buffer supplemented with 1 M salt, followed with a further wash with 5 column volumes of Lysis buffer. The protein was eluted with 20 mM glutathione (pH 7.0). The GST tag was then cleaved using recombinant HRV-3C protease.

Size exclusion chromatography was performed on Superdex 75 (26/60) column (GE healthcare) attached to an Akta prime system at 4°C. The absorbance of the eluate was monitored at 280 nm throughout. The column was equilibrated with phosphate buffer (40 mM sodium phosphate, 100 mM NaCl, 1 mM DTT, 5% glycerol, pH 7.5). 5 mL of protein (after affinity chromatography) was loaded onto the column using a 5 mL injection loop. Each fraction from within the peak of the UV trace was analysed by SDS-PAGE.

eIF4e (35-216)

pETsumo-eIF4e was transformed in to BL21 (DE3) pLysS Star cells and over-expressed by auto-induction. Overnight cultures were grown in minimal media (Table ESI 1) and 400 µL of this overnight culture was used to inoculate 400 mL auto-induction media (Table ESI 2). The culture was grown for 4 hours at 37°C with aeration, then reduced to 20°C with aeration until no more cell growth was apparent (no more increase in OD). Cells were harvested and resuspended in 15 mL Lysis buffer per 400 mL culture.

Table ESI 1. Composition of minimal media for autoinduction

Solution	Composition	Volume (µL)
H_2O	H_2O	4775
$MgSO_4$	1 M $MgSO_4$	10
1000x trace elements	50 mM $FeCl_3$<br>20 mM $CaCl_2$<br>10 mM $MnCl_2$<br>2 mM $CoCl_2$<br>2 mM $CuCl_2$<br>2 mM $NiCl_2$<br>2 mM Na_2MoO_4<br>2 mM H_3BO_3	1
40 % glucose	40% glucose (w/v)	62.5
25% aspartate	25% aspartate (w/v)	50
50x M	1.25 M Na_2HPO_4<br>1.25 M KH_2PO_4<br>2.5 M NH_4Cl<br>0.25 M Na_2SO_4	100

Table ESI 2. Composition of auto induction media

Solution	Composition	Volume (mL)
ZY media	5 g/L yeast extract<br>10 g/L tryptone	383
1 M $MgSO_4$	1 M $MgSO_4$	0.8
1000x trace elements	See table	1
50x 5052	25% glycerol (w/v)<br>2.5% glucose (w/v)<br>10% lactose (w/v)	8
50x M	See table	8

The fusion protein was purified by Ni^{2+} sepharose HisTrap HP column (GE Healthcare). Columns were washed with 5 column volumes of water, charged with 0.1 M $NiSO_4$ and equilibrated with 5 column

volumes of Lysis buffer before loading the of filtered supernatant. The filtered supernatant was passed through the column and the flow through collected. The column was then washed with 5 column volumes of Lysis buffer. Elution was carried out by increasing concentration of imidazole in Lysis buffer (80-400 mM). All wash fractions were collected and analysed by SDS-PAGE.

Size exclusion chromatography was performed on Superdex 75 (26/60) column (GE healthcare) attached to an Akta prime system at 4°C. The absorbance of the eluate was monitored at 280 nm throughout. The column was equilibrated with phosphate buffer (40 mM sodium phosphate, 200 mM NaCl, 1 mM DTT, 5% glycerol, pH 7.5). 5 mL of protein sample (after affinity chromatography) was loaded onto the column using a 5 mL injection loop. Each fraction from within the peak of the UV trace was analysed by SDS-PAGE.

PEPTIDES

HIF-1α CTAD Peptides

The HIF-1α *C*-terminal transactivation domain (CTAD) peptides were purchased from Proteogenix, France. The sequences are shown below.

Ac-SMDESGLPQLTSYDCEVNAPIQGSRNLLQGEELLRALDQVN-NH_2

The labelled analogue has the same sequence with an additional aminohexanoic acid (Ahx) residue was the *N*-terminus which was functionalised with fluorescein isothiocyanate (FITC).

FITC-Ahx-SMDESGLPQLTSYDCEVNAPIQGSRNLLQGEELLRALDQVN-NH_2

Peptide Synthesis

Additional peptides were synthesized by manual or automated Fmoc solid phase peptide synthesis methods on Rink Amide MBHA resin. For each coupling step 5 eq. of the Fmoc-Amino acid in DMF was added to the resin along with 5 eq. of HCTU and 5 eq. of DIPEA in DMF and mixed for 2 hours followed by washing the resin 3 times for 2 minutes with DMF. For the Fmoc deprotection the resin was treated with an excess of 20% Piperidine in DMF 5 times for 2 minutes each followed by washing 5 times with DMF for 2 minutes each.

Acetylation

The resin bound peptide was treated with 10 eq. of acetic anhydride and 10 eq. of DIPEA and mixed overnight.

Fluorescein Labelling

Fmoc-amino hexanoic acid was coupled to the resin bound peptide and deprotected as before and it was then treated with fluorescein isothiocyanate (1.2 eq.) overnight in the minimum volume of 12:7:5 pyridine/DMF/CH_2Cl_2. The pyridine was distilled over CaH_2 immediately before use.

Deprotection and Cleavage

The resin was washed with DMF 3 times and DCM 3 times prior to cleavage and deprotection. The peptides were globally deprotected and cleaved from the resin with TFA/thioanisole/water/phenol/EDT (83:5:5:5:2) [Reagent K], concentrated *in vacuo*, precipitated with ice cold ether, washed 3 times with ice cold ether and dried *in vacuo*. Peptides were then purified by preparative HPLC and lyophilised.

HIF-1 *C*-terminal Helix (Helix 3) Peptide

Ac-GTEELLRALDQVNAAG-NH_2

LC-MS *m/z* (ES) 1697.7 $[M+H]^+$, 849.8 $[M+2H]^{2+}$; HPLC r.t. 3.48 min (5-95% H_2O-MeCN + 0.1% TFA, 5.40 min, 0.5ml/min, Ascentis Peptide). HRMS Found: 849.4462, 1697.8843; $C_{70}H_{120}N_{22}O_{26}$ requires $[M+2H]^{2+}$ 849.4444, $[M+H]^+$ 1697.8851

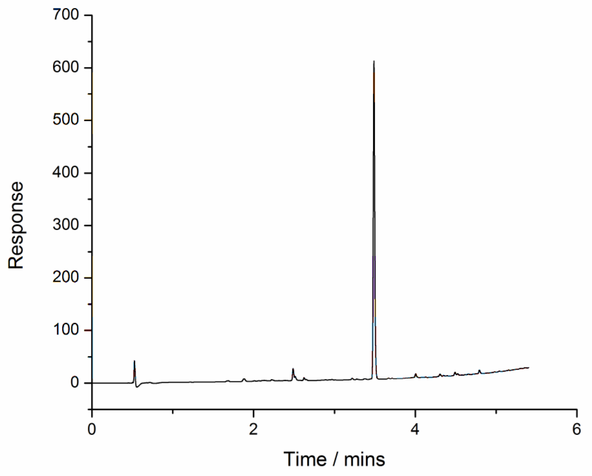

eIF4g Peptides

Ac-RYDREFLLGFQ-NH_2

LC-MS *m/z* (ES) 1484.7 $[M+H]^+$, 743.0 $[M+2H]^{2+}$; HPLC r.t. 2.49 min (5-95% H_2O-MeCN + 0.1% TFA, 5.40 min, 0.5ml/min, Ascentis Peptide). HRMS Found: 742.8860; $C_{69}H_{101}N_{19}O_{18}$ requires $[M+2H]^{2+}$ 742.8786.

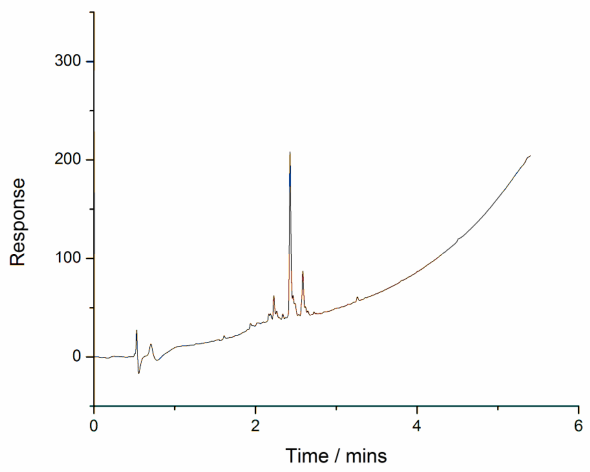

Fluorescein-Ahx-RYDREFLLGFQ-NH$_2$

LC-MS *m/z* (ES) 1945.6 $[M+H]^+$, 973.4 $[M+2H]^{2+}$, 649.7 $[M+3H]^{3+}$; HRMS Found: 1966.8350; $C_{94}H_{121}N_{21}NaO_{23}S$ requires $[M+Na]^+$ 1966.8557.

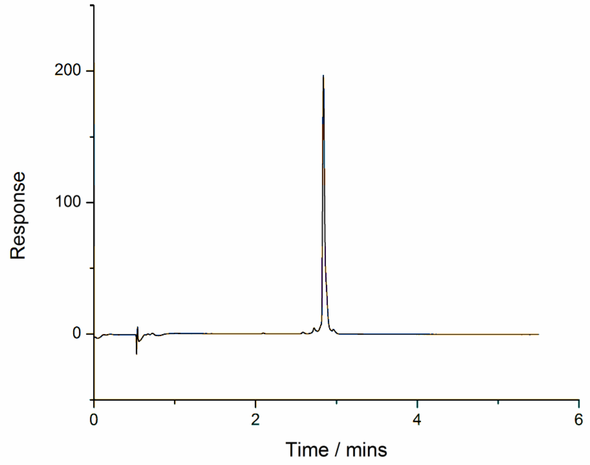

FLUORESCENCE ANISOTROPY ASSAYS

Determination of the Binding of Labelled HIF-1α Peptide to p300 Protein

Protein was serially diluted into a solution of labelled peptide (80 nM) and the plates incubated for 30 minutes at room temperature. Each experiment was run in triplicate and the fluorescence anisotropy measured using a EnVision 2103 MultiLabel plate reader (Perkin Elmer) with excitation at 480 nm and emission at 535 nm (5 nm bandwidths). The Intensity was calculated for each point using Eq. 1 and used to calculated anisotropy using Eq. 2. From a plot of anisotropy against protein

concentration, the minimum and maximum anisotropies were obtained using a logistic sigmoidal fit in OriginPro 8.6.

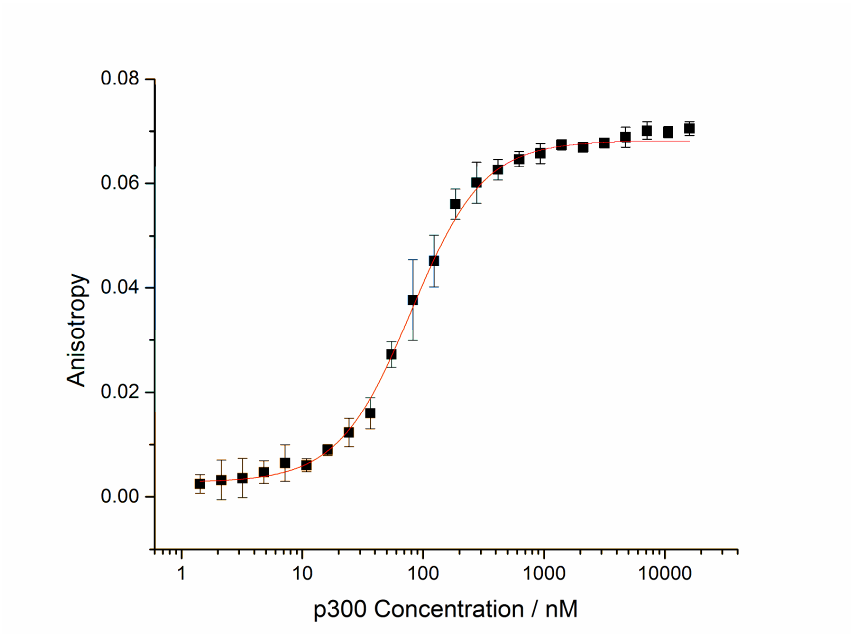

Figure ESI 1. Determination of minimum and maximum anisotropy (80 nM FITC-HIF-1α CTAD, 40 mM sodium phosphate, 100 mM NaCl, 1 mM DTT, 5% glycerol). Error bars represent the standard deviation of 3 repeats.

These values were used to convert anisotropy into amount of bound labelled peptide using Eq. 3. This data was then fitted using Eq. 4 in OriginPro 8.6 to determine the dissociation constant, K_D.

$$I = (2PG) + S \qquad \text{(Eq.1)}$$

$$r = \frac{S-PG}{1} \qquad \text{(Eq. 2)}$$

$$L_b = \frac{r - r_{min}}{\lambda(r_{max} - r) + r - r_{min}} \qquad \text{(Eq. 3)}$$

$$y = \frac{(K_D + x + [FL]) - \sqrt{[\{(K_D + x + [FL])\}^2 - 4x[FL]]}}{2} \qquad \text{(Eq. 4)}$$

R= anisotropy, I= total intensity, P=perpendicular intensity, S= parallel intensity, G = an instrument factor set to 1, L_b = fraction ligand bound, $\lambda = I_{bound}/I_{unbound}$ = 1, [FL] = concentration of fluorescent peptide, K_D = dissociation constant, y = L_b multiplied by [FL], x = protein concentration

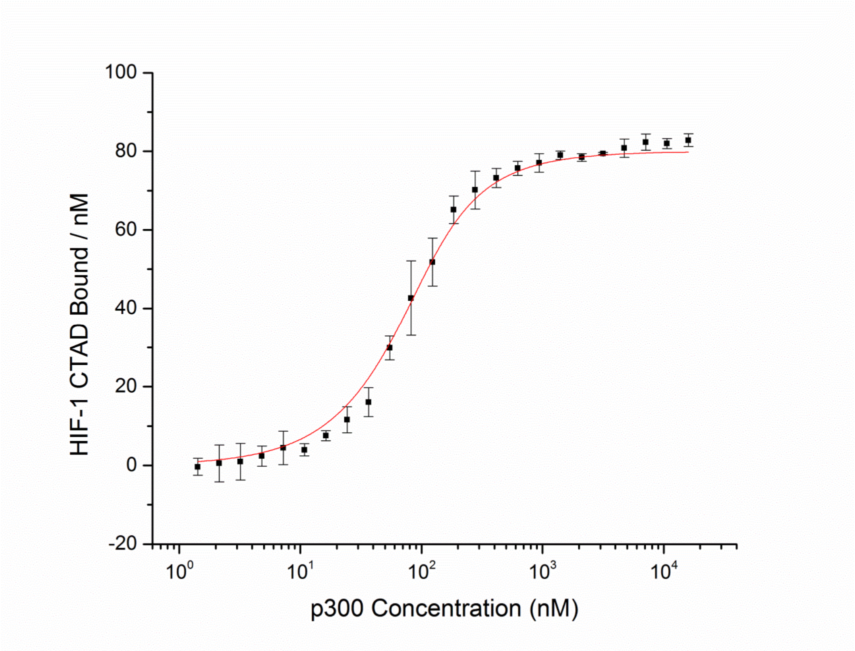

Figure ESI 2. Direct titration of p300 into FITC-HIF-1α C-TAD followed by fluorescence anisotropy K_D = 37.4 ± 4.4 nM (80 nM FITC-HIF-1α CTAD, 40 mM sodium phosphate, 100 mM NaCl, 1 mM DTT, 5% glycerol). Error bars represent the standard deviation of 3 repeats.

Competition Assays

Compounds to be tested were serially diluted across a 384 well plate in buffer containing the minimum DMSO (final concentration <5%) before labelled peptide and protein were added sequentially. In parallel, a control experiment was performed in which no labelled peptide was added and the volume made up with additional buffer. Each experiment was performed in triplicate using a EnVision 2103 MultiLabel plate reader (Perkin Elmer) with excitation at 480 nm and emission at 535 nm (5 nm bandwidths) at 25 °C. Intensity and anisotropy were calculated as above using Eq. 1 and Eq. 2 respectively. Plots of anisotropy against compound concentration were fitted to a logistic sigmoidal dose response model to determine IC_{50} values.

A positive control was performed with the unlabelled version of the labelled tracer peptide which is shown below.

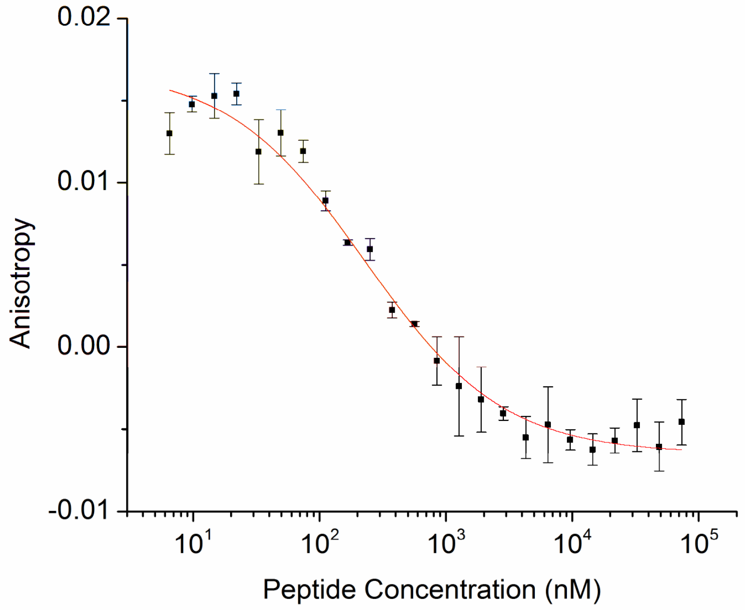

Figure ESI 3. competition titration of p300/HIF-1α* with HIF-1α CTAD followed by fluorescence anisotropy IC_{50} = 228 ± 25 nM (80 nM FITC-HIF-1α CTAD, 0.1 µM p300, 40 mM sodium phosphate, 100 mM NaCl, 1 mM DTT, 5% glycerol, 0.1% triton). Error bars represent the standard deviation of 3 repeats.

Determination of the Binding of Labelled eIF4G Peptide to eIF4E Protein

Development of a fluorescence anisotropy binding assay for eIF4E/eIF4G was performed as for the HIF-1a/p300 interaction described above.

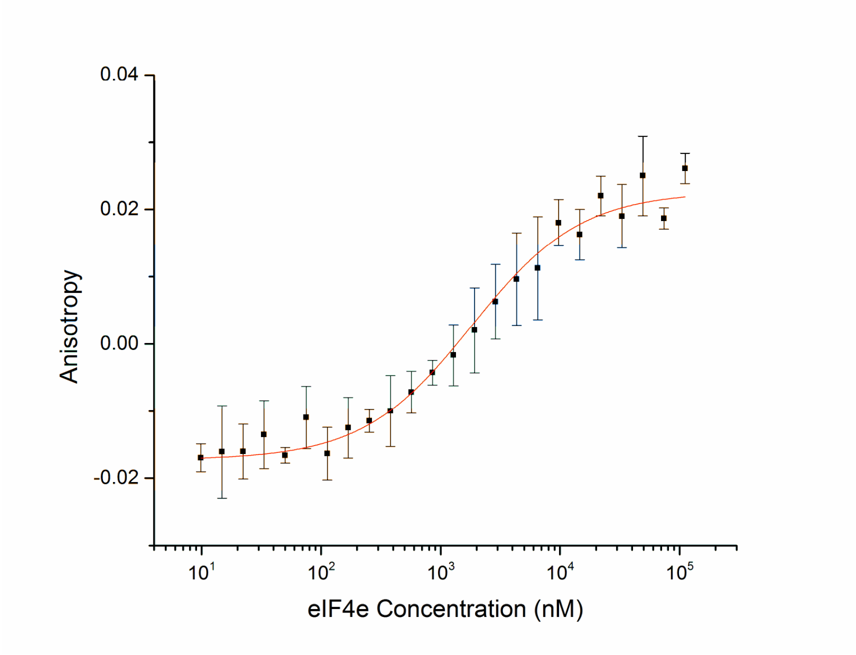

Figure ESI 4. Direct titration of eIF4E into FITC-eIF4G followed by fluorescence anisotropy K_D = 1.76 ± 0.17 µM (80 nM FITC-eIF4g, 40 mM sodium phosphate, 200 mM NaCl, 1 mM DTT, 5% glycerol, 0.1% triton). Error bars represent the standard deviation of 3 repeats.

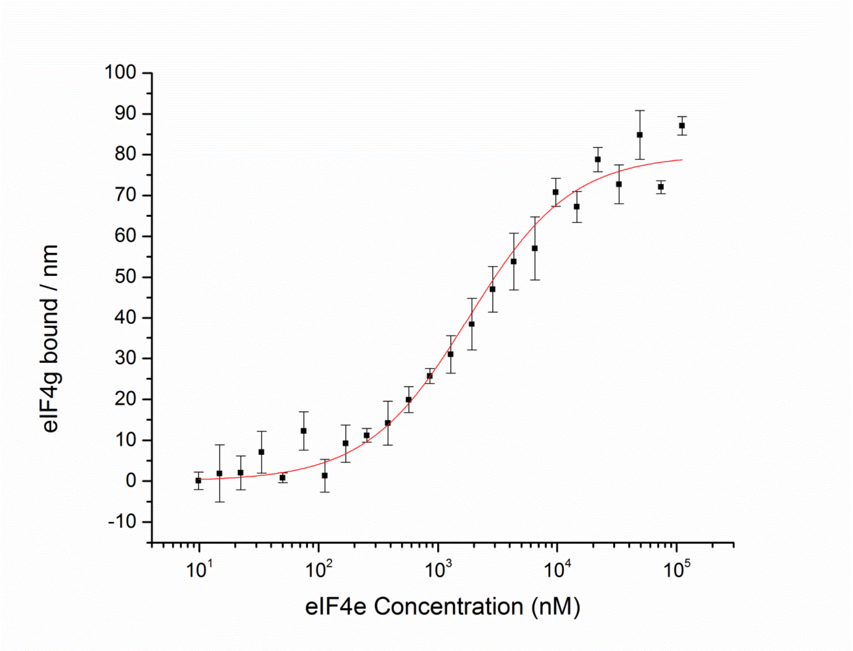

Figure ESI 5. Direct titration of eIF4E into FITC-eIF4G followed by fluorescence anisotropy K_D = 1.76 ± 0.17 µM (80 nM FITC-eIF4g, 40 mM sodium phosphate, 200 mM NaCl, 1 mM DTT, 5% glycerol, 0.1% triton). Error bars represent the standard deviation of 3 repeats.

The assay was validated for identification of competitive inhibitors through competition titration with unlabelled peptide as described for the HIF-1α/p300 interaction above.

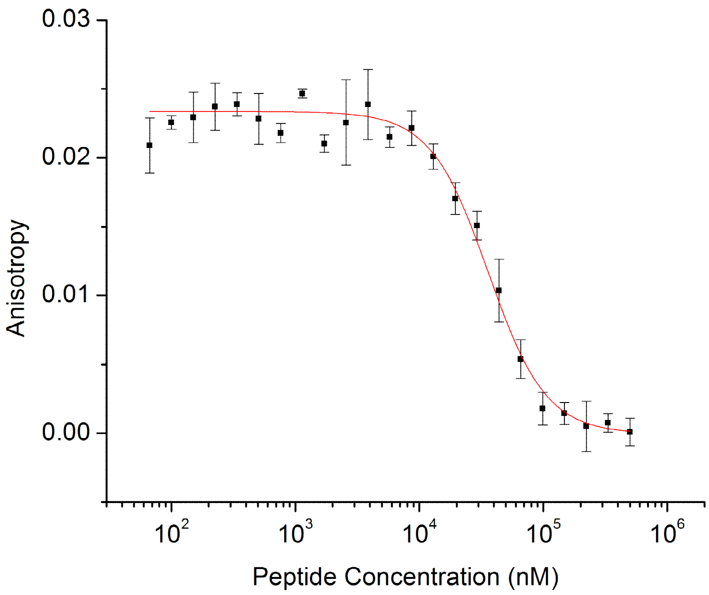

Figure ESI 6. competition titration of eiF4E/FITC-eIF4G with eIF4G followed by fluorescence anisotropy IC_{50} = 36 ± 4.5 µM (80 nM FITC-eIF4G, 3 µM eIF4e, 40 mM sodium phosphate, 200 mM NaCl, 1 mM DTT, 5% glycerol, 0.1% triton). Error bars represent the standard deviation of 3 repeats.

TRYPTOPHAN FLUORESCENCE BINDING ASSAY

Spectra were recorded on a FluoroMax-3 spectrofluorometer (Horiba Jobin Yvon Inc.) in 10 mm quartz fluorimeter cell at 25 °C with 4 nm excitation and 4 nm emission slit widths. Samples were excited at 295 nm and emission recorded between 200 and 400 nm. Compound solutions were prepared in DMSO and 4 µL of stock solution added to 400 µL of 30 µM protein in phosphate buffer to give final concentrations of between 200 µM and 0 µM. Samples were equilibrated for 30 minutes before analysis. Absolute values of fluorescence were recorded at 335 nm and plotted against compound concentration.

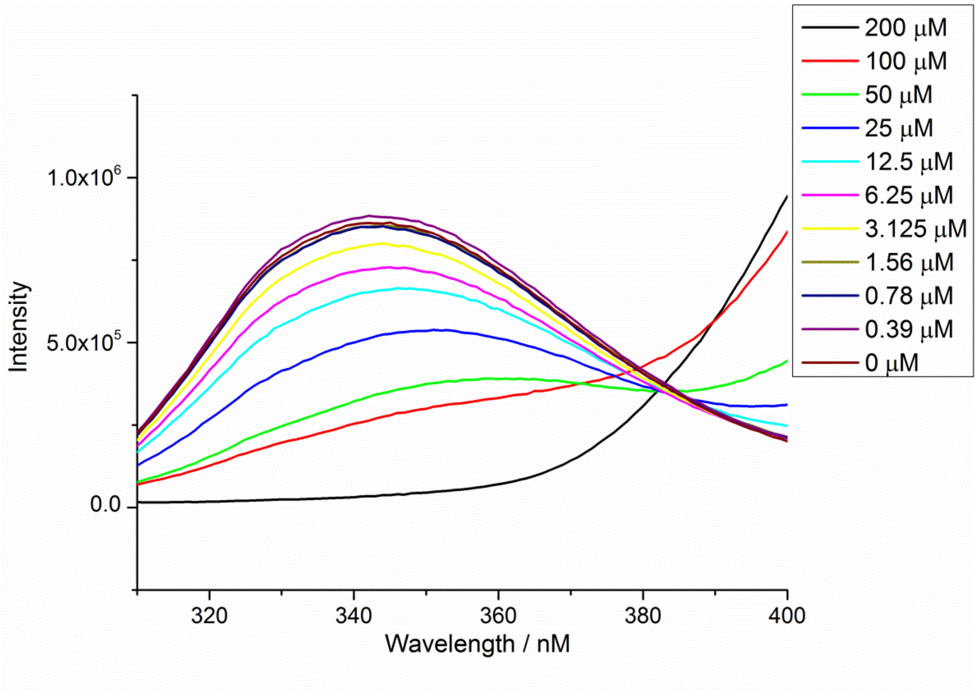

Figure ESI 7 – Quenching of fluorescence with increasing compound concentration.

During data analysis we observed complete quenching of the fluorescence signal upon increasing compound concentration resulting in a zero emission at 335 nm. Further analysis of the optical properties of compound **3** revealed a strong absorbance band spanning the excitation and emission wavelengths used in this experiment (295-335 nm) resulting in a significant inner filter effect. The calculated extinction coefficients for the compound were approximately $2x10^6$ M^{-1} $cm^{-1}$and $1x10^6$ M^{-1} cm^{-1} at 335 nm and 295 nm respectively.

DOCKING

The protein structure for p300 (PDB ID: 1L8C) was prepared using the protein preparation wizard within Maestro (Schrodinger) and the docking grid prepared by selecting the binding groove of the *C*-terminal helix of the HIF-1α CTAD using Glide (Schrodinger).

Conformer libraries of compounds were generated using Omega (Openeye Scientific) and prepared for docking using Ligprep (Schrodinger). Docking was performed using Glide (Schrodinger) allowing flexible ligands but penalizing non-planar amide bonds.

COMPETITION ASSAYS CURVES

Error bars represent the standard deviation of three repeats in all cases.

Compound 3

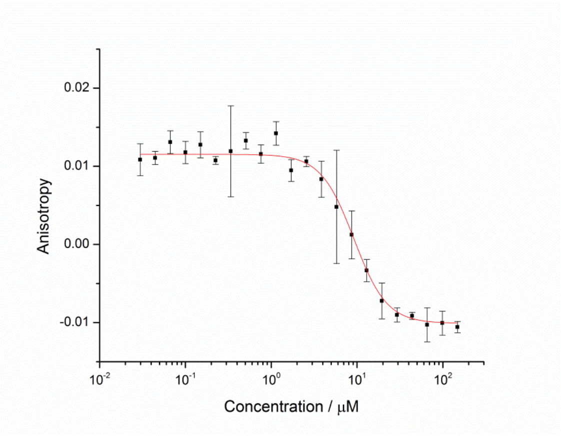

Compound 4

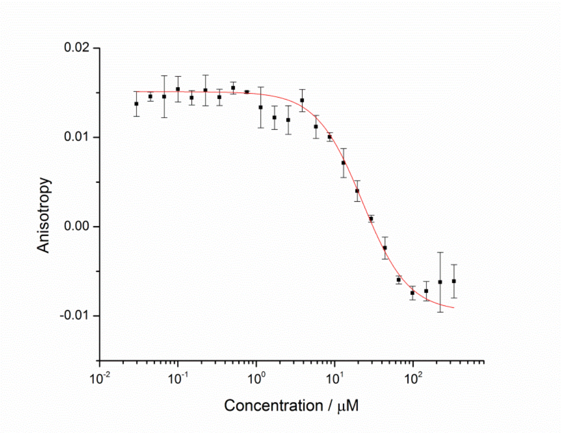

Compound 5

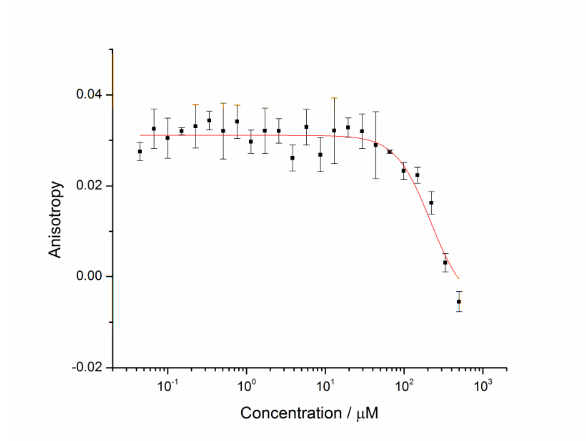

Compound 6

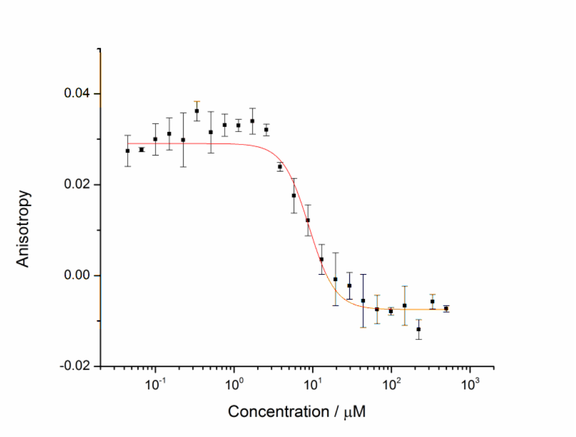

Compound 7

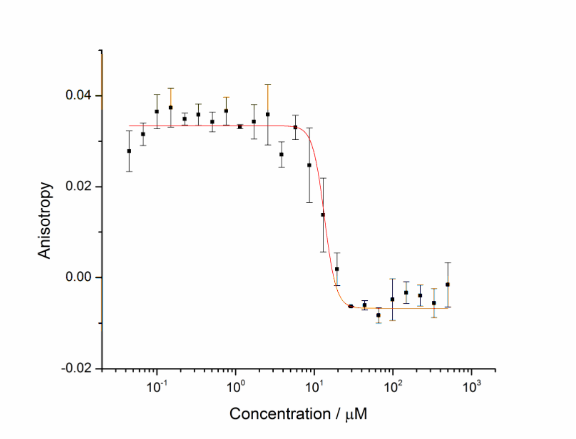

Compound 8

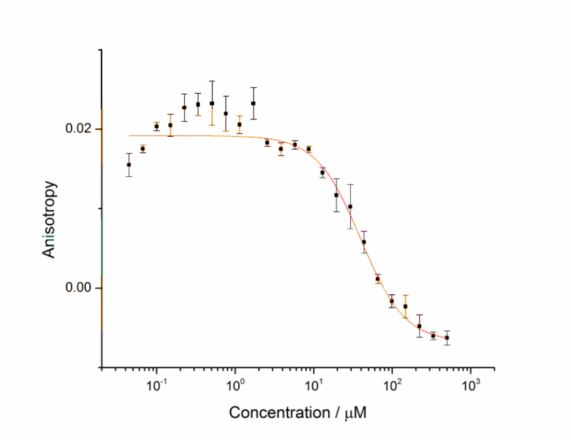

Compound 9

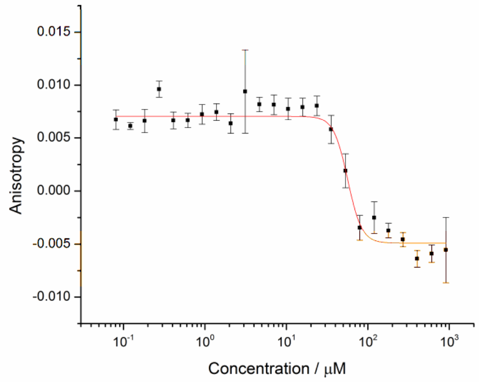

Compound 10

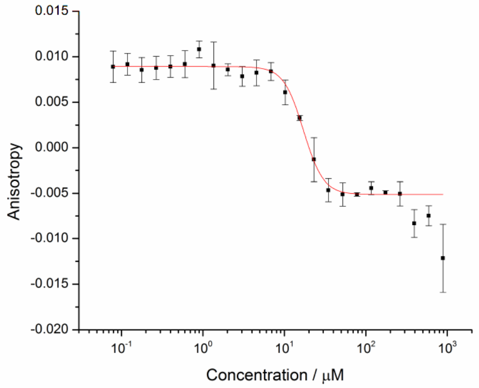

Compound 11

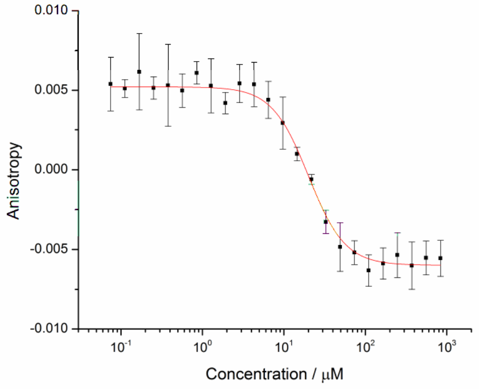

Compound 12

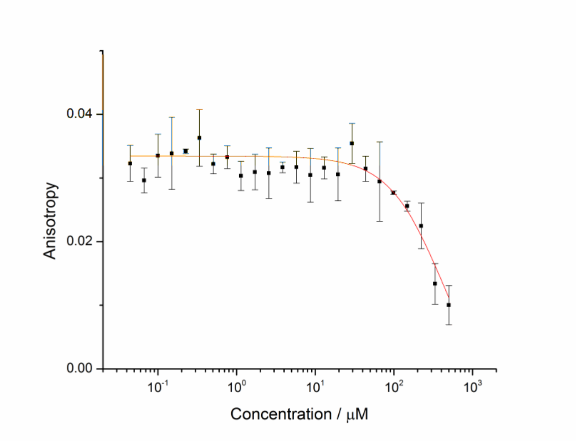

Compound 13

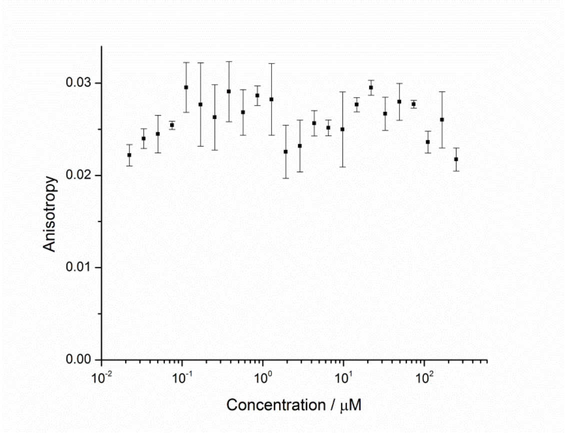

C-terminal Helix (Helix 3) Peptide

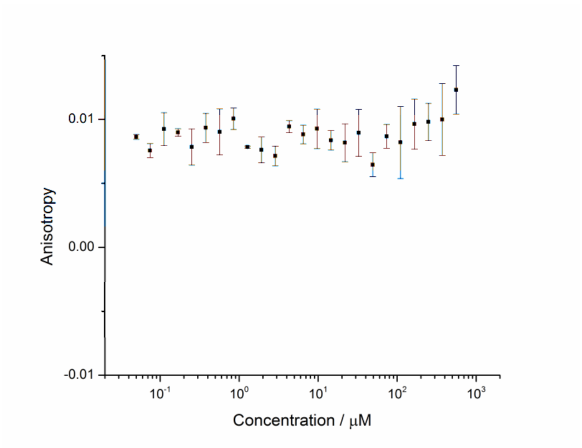

Compound 2

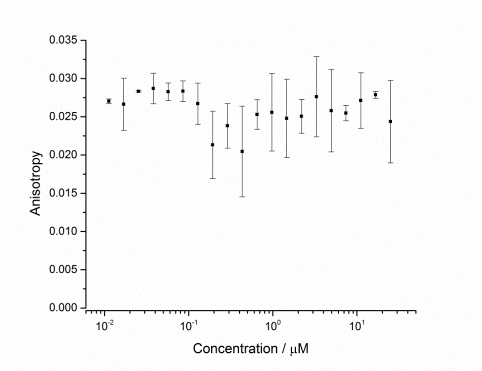

0.035
0.030
0.025
0.020
0.015
0.010
0.005
0.000
Anisotropy
10^{-2}
10^{-1}
10^{0}
10^{1}
Concentration / μM

Spectra of Final Compounds

Compound 2 – ^{1}H NMR, 500 MHz, $CDCl_3$

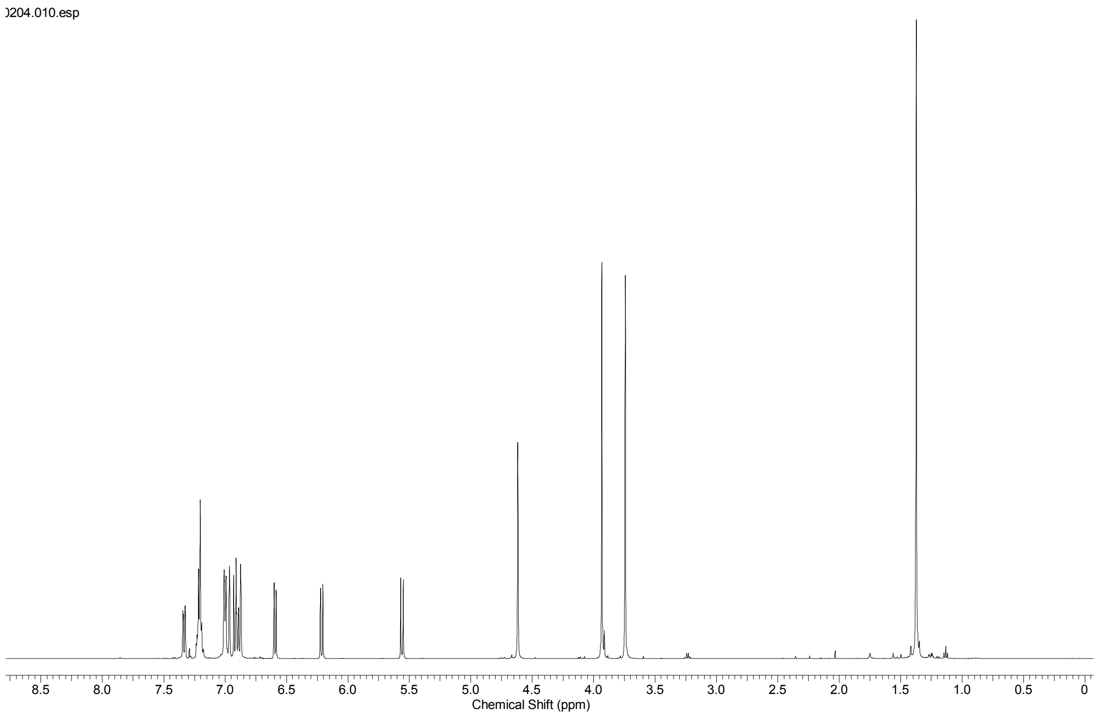

Compound 3 - ^{1}H NMR, 500 MHz, d_6-DMSO

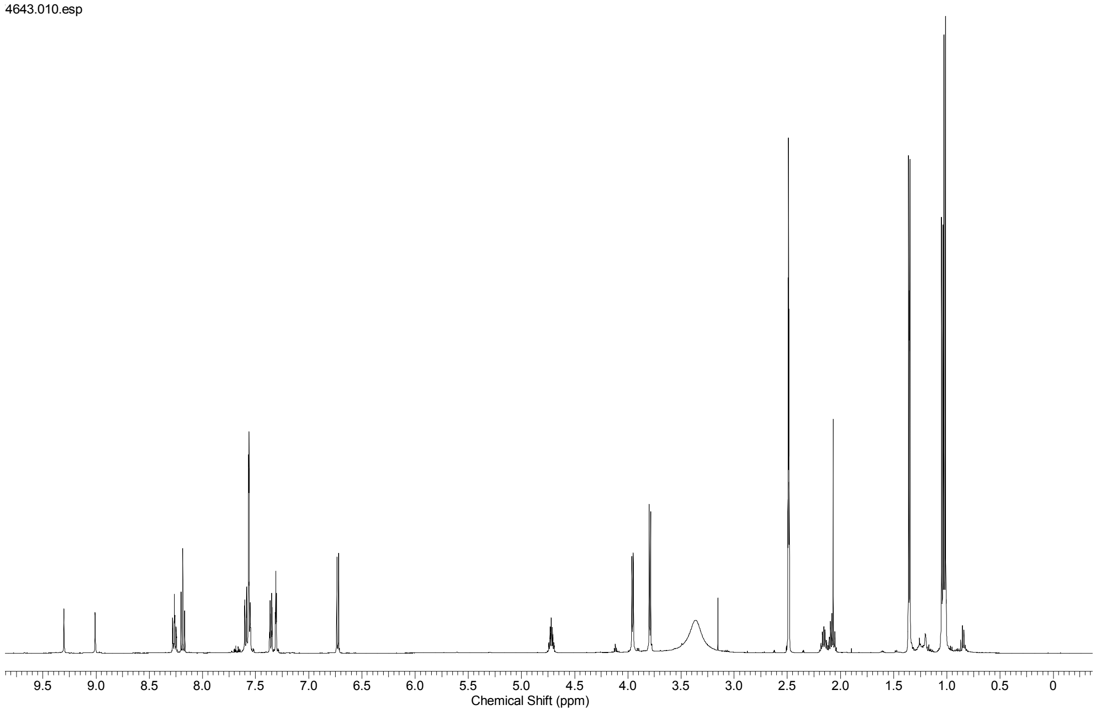

Compound 4- ^{1}H NMR, 500 MHz, d_6-DMSO

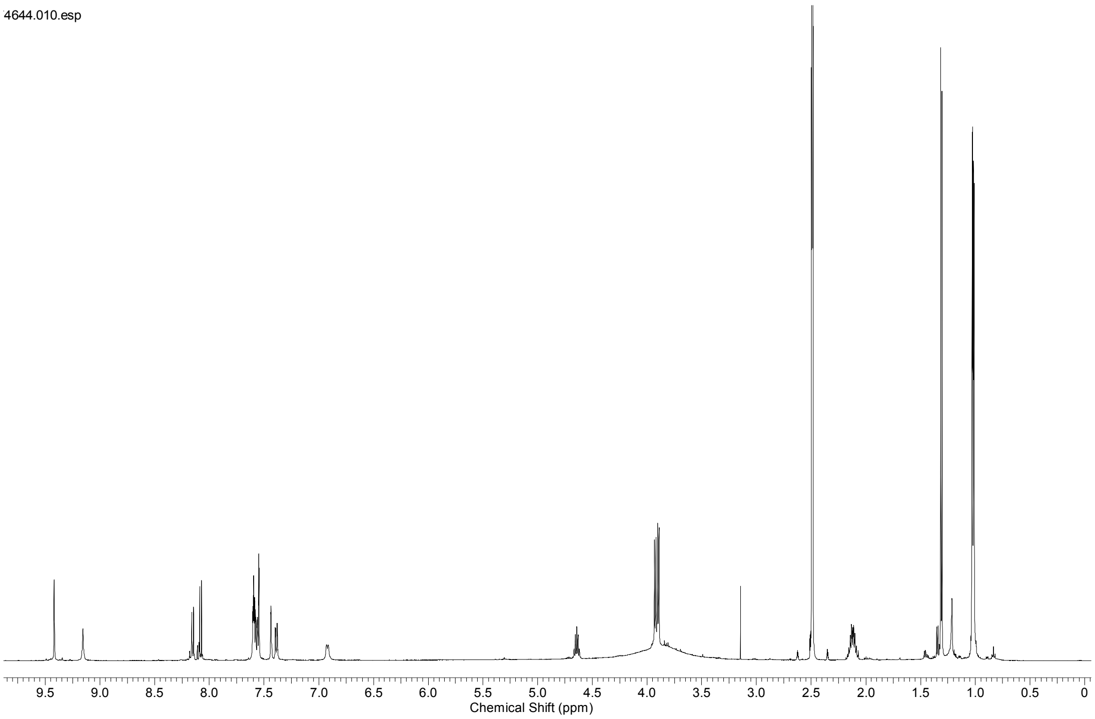

Compound 5- ^{1}H NMR, 500 MHz, d_6-DMSO

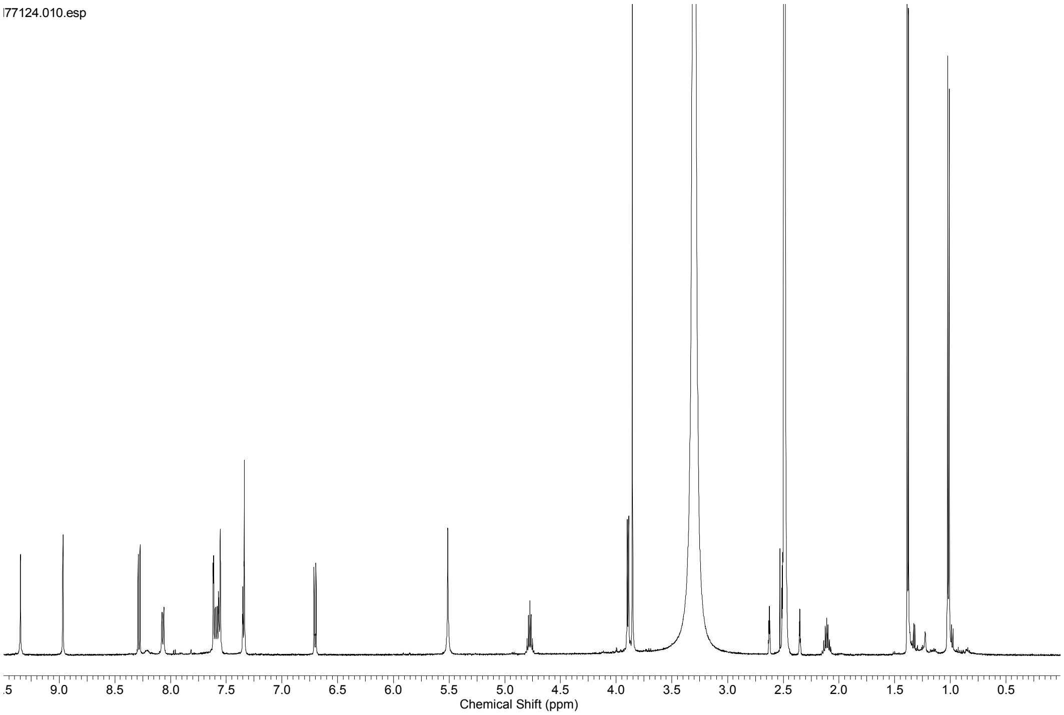

Compound 6 - ^{1}H NMR, 500 MHz, d_6-DMSO

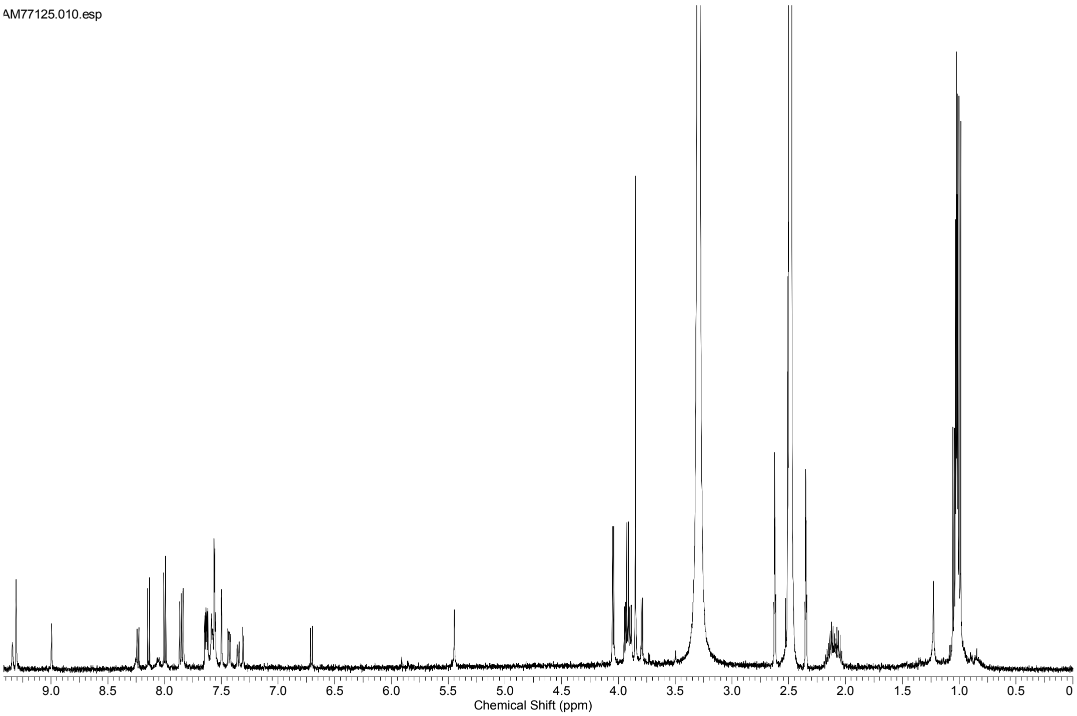

Compound 7- ^{1}H NMR, 500 MHz, d_6-DMSO

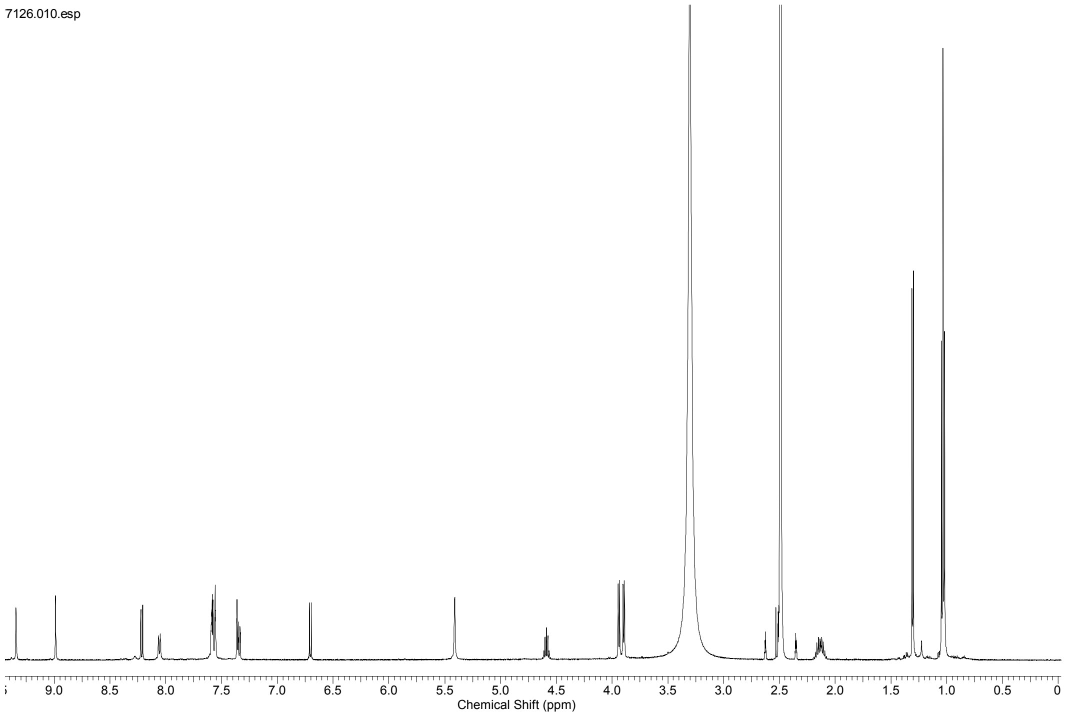

Compound 8- ^{1}H NMR, 500 MHz, d_6-DMSO

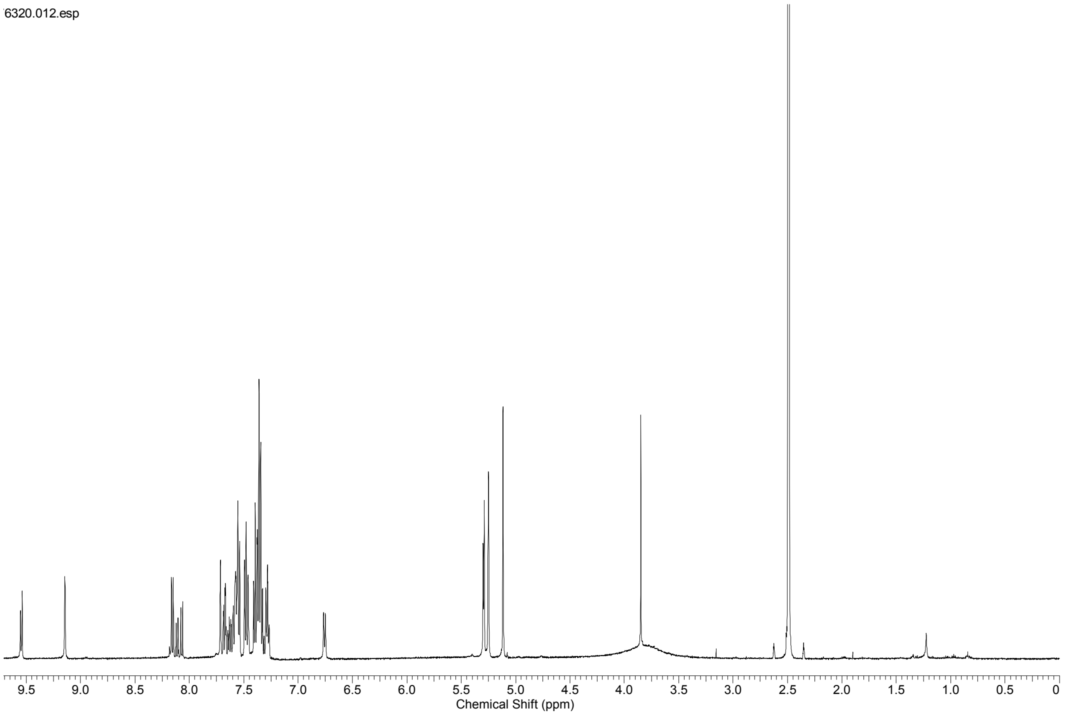

Compound 9- ^{1}H NMR, 500 MHz, d_6-DMSO

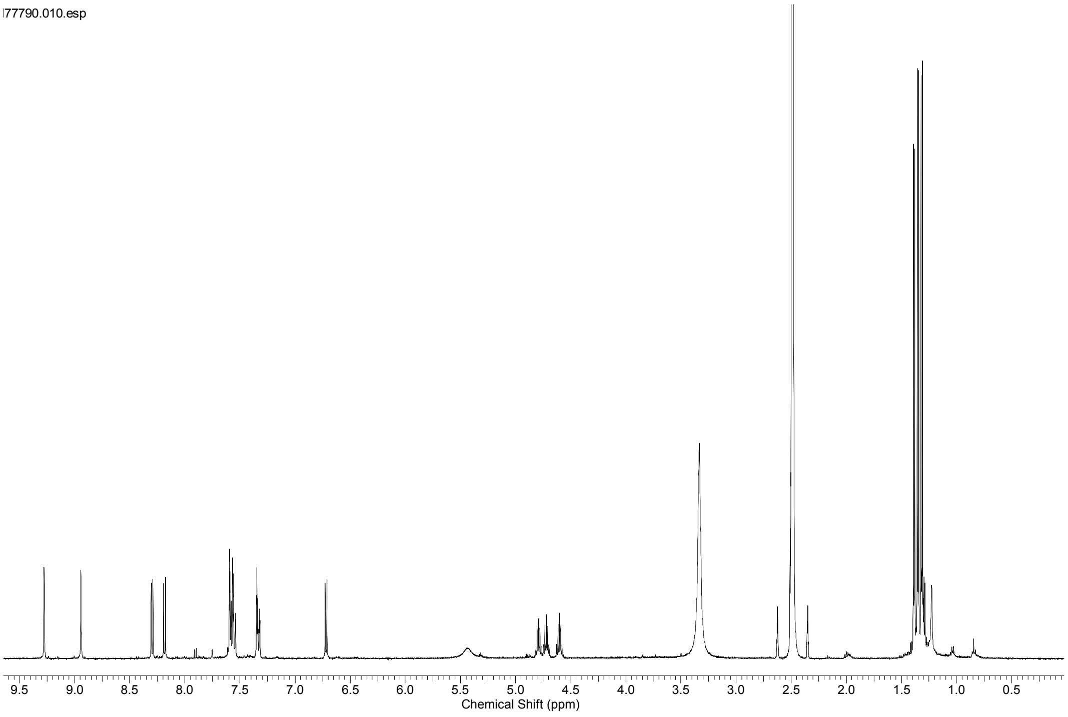

Compound 10- ^{1}H NMR, 500 MHz, d_6-DMSO

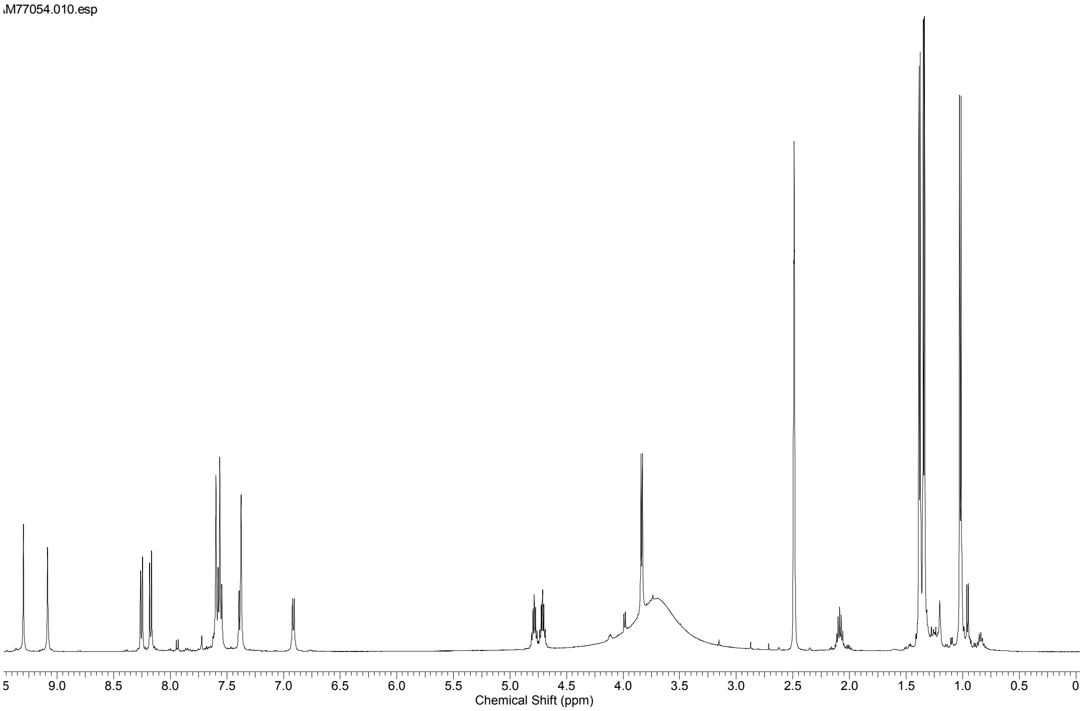

Compound 11- ^{1}H NMR, 500 MHz, d_6-DMSO

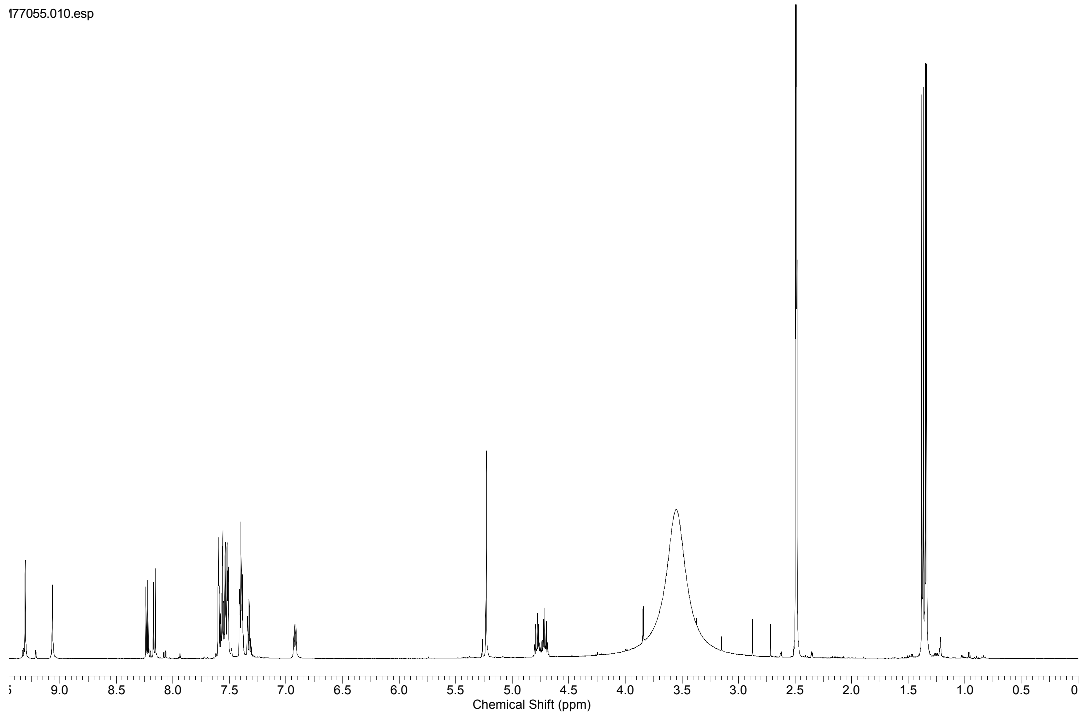

Compound 12- ^{1}H NMR, 500 MHz, d_6-DMSO

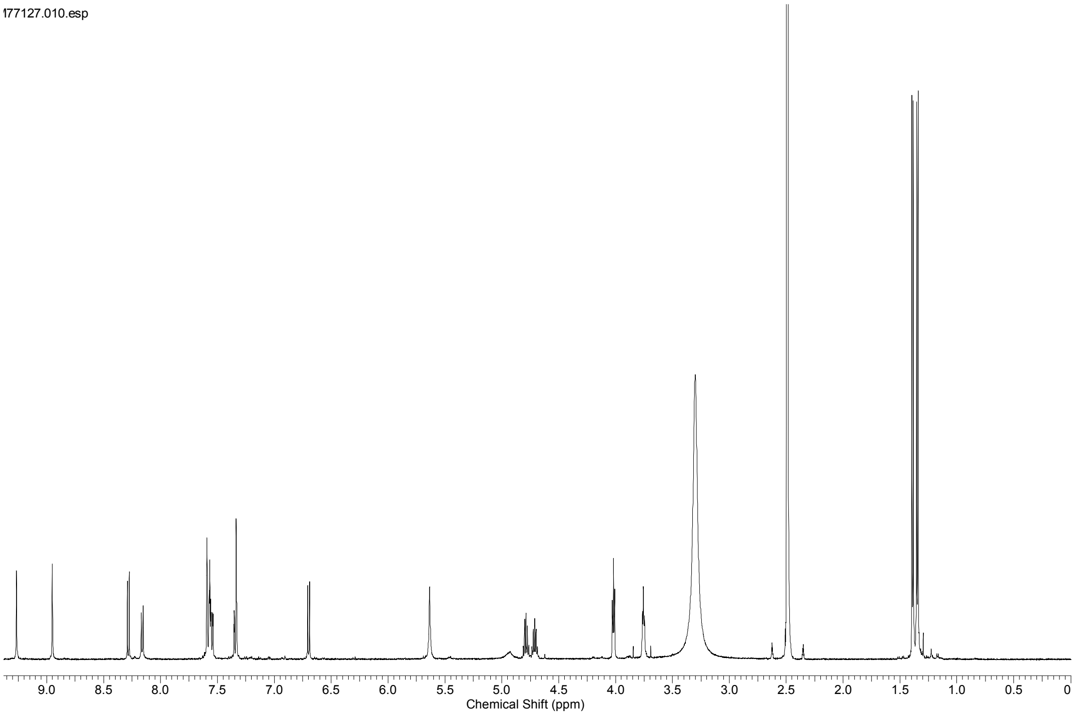

References

[1] J. Plante, F. Campbell, B. Malkova, C. Kilner, S. L. Warriner, A. J. Wilson, *Org. Biomol. Chem.* **2008**, *6*, 138-146.

[2] N. S. Murphy, P. Prabhakaran, V. Azzarito, J. P. Plante, M. J. Hardie, C. A. Kilner, S. L. Warriner, A. J. Wilson, *Chem. Eur. J.* **2013**, *19*, 5546-5550.

[3] F. Campbell, J. P. Plante, T. A. Edwards, S. L. Warriner, A. J. Wilson, *Org. Biomol. Chem.* **2010**, *8*, 2344-2351.

[4] R. L. Farmer, M. M. Biddle, A. E. Nibbs, X. Huang, R. C. Bergan, K. A. Scheidt, *ACS Medicinal Chemistry Letters* **2010**, *1*, 400-405.

[5] J. Mun, A. A. Jabbar, N. S. Devi, Y. Liu, E. G. Van Meir, M. M. Goodman, *Bioorg. Med. Chem.* **2012**, *20*, 4590-4597.
